# Deficiency in the glycosyltransferase Gcnt1 increases susceptibility to tuberculosis through a mechanism involving neutrophils

**DOI:** 10.1038/s41385-020-0277-7

**Published:** 2020-03-13

**Authors:** Kaori L. Fonseca, Ana Raquel Maceiras, Rita Matos, Luisa Simoes-Costa, Jeremy Sousa, Baltazar Cá, Leandro Barros, Ana Isabel Fernandes, Stefan Mereiter, Ricardo Reis, Joana Gomes, Gustavo Tapia, Paula Rodríguez-Martínez, Montse Martín-Céspedes, Sergo Vashakidze, Shota Gogishvili, Keti Nikolaishvili, Rui Appelberg, Fátima Gärtner, Pedro N. S. Rodrigues, Cristina Vilaplana, Celso A. Reis, Ana Magalhães, Margarida Saraiva

**Affiliations:** 1 i3S—Instituto de Investigação e Inovação em Saúde, Universidade do Porto, Porto, Portugal; 2 IBMC—Instituto de Biologia Molecular e Celular, Universidade do Porto, Porto, Portugal; 3 Programa de Pós-Graduação Ciência para o Desenvolvimento (PGCD), Instituto Gulbenkian de Ciência (IGC), Oeiras, Portugal; 4 ICBAS—Instituto de Ciências Biomédicas Abel Salazar, University of Porto, Porto, Portugal; 5 IPATIMUP—Instituto de Patologia e Imunologia Molecular da Universidade do Porto, Porto, Portugal; 6 CDP-Centro de Diagnóstico Pneumológico do Porto, Porto, Portugal; 7 UAB—Pathology Department, Universitat Autònoma de Barcelona, Hospital Universitari Germans Trias i Pujol, Barcelona, Spain; 8 National Center for Tuberculosis and Lung Diseases (NCTLD), Tbilisi, Georgia; 9 UAB—Experimental Tuberculosis Unit. Universitat Autònoma de Barcelona, CIBER Enfermedades Respiratorias. Fundació Institut d’Investigació en Ciències de la Salut Germans Trias i Pujol, Barcelona, Spain; 10 FMUP—Faculdade de Medicina da Universidade do Porto, Porto, Portugal

## Abstract

Modulation of immunity and disease by glycans is increasingly recognized. However, how host glycosylation shapes and is shaped by tuberculosis remains poorly understood. We show that deficiency in the glucosaminyl (N-acetyl) transferase 1 (Gcnt1), a key enzyme for core-2 *O*-glycans biosynthesis, drives susceptibility to *Mycobacterium tuberculosis* infection. The increased susceptibility of *Gcnt1* deficient mice was characterized by extensive lung immune pathology, mechanistically related to neutrophils. Uninfected *Gcnt1* deficient mice presented bone marrow, blood and lung neutrophilia, which further increased with infection. Blood neutrophilia required Gcnt1 deficiency in the hematopoietic compartment, relating with enhanced granulopoiesis, but normal cellular egress from the bone marrow. Interestingly, for the blood neutrophilia to translate into susceptibility to *M. tuberculosis* infection, Gnct1 deficiency in the stroma was also necessary. Complete Gcnt1 deficiency associated with increased lung expression of the neutrophil chemoattractant CXCL2. Lastly, we demonstrate that the transcript levels of various glycosyltransferase-encoding genes were altered in whole blood of active tuberculosis patients and that sialyl Lewis x, a glycan widely present in human neutrophils, was detected in the lung of tuberculosis patients. Our findings reveal a previously unappreciated link between Gcnt1, neutrophilia and susceptibility to *M. tuberculosis* infection, uncovering new players balancing the immune response in tuberculosis.

## Introduction

The host immune system has evolved to respond to infection whilst avoiding tissue damage. Deregulation of protective immune responses to *Mycobacterium tuberculosis* (*Mtb*) often associates with tissue pathology.^1^ For example, an inadequate recruitment of neutrophils to *Mtb*-infected lungs may cause severe tissue damage. Exacerbated neutrophilia is thus linked with increased severity of infection in mouse^2,3^ and man.^4^ Understanding the factors that control or disrupt protective immune networks during tuberculosis (TB) is therefore paramount for the design of more efficient strategies and to inform on potential TB-associated side effects of immune therapies to other diseases.

The importance of glycosylation in innate and acquired immune responses is emerging.^5^ Glycosylation regulates leukocyte recruitment from the vasculature to most tissues, through the modulation of interactions between endothelial selectins and their ligands in leukocytes.^6,7^ Deficiency in several glycosyltransferases involved in the biosynthesis of functional selectin ligands, as *Galnt1*, *Gcnt1*, *B4galt1*, *Fut4*, *Fut7* and *St3gal4*, results in defective leukocyte recruitment^7–9^ and in homeostatic alterations, namely pronounced blood neutrophilia.^10,11^ Selectin ligands are commonly decorated with *O*-glycans bearing a terminal sialyl Lewis x (sLe^x^) (sialic acid α2,3Galβ1–4 (Fucα1,3) GlcNAc-R) epitope.^6^ Extensive *O*-glycosylation is also characteristic of transmembrane or secreted gel-forming mucins,^12^ important components of the lung mucus. Mucins play key functions as surface receptors for the binding of several adhesion molecules, mediators of the interaction with pathogens, and local modulators of inflammatory responses.^13,14^ Pathogens, as *Helicobacter pylori*, may modulate the expression, turnover and glycosylation of host mucins.^15–18^ How *O*-glycosylation contributes to host protection in TB is largely unknown. Deficiency in the α1,3-fucosyltransferase Fut7 or Fut4/Fut7 double deficiency impaired T cell homing to the lymph nodes during *Mtb* experimental infection, but the traffic of effector T cells to the lung was normal.^19^ Both *Fut7* deficient (^−/−^) and *Fut4*^−/−^/*Fut7*^−/−^ mice were initially shown to resist normally to low dose aerosol infection,^19^ but a shorter survival of these mice upon infection was reported in a later study.^20^

Here, we reveal that core-2 GlcNAc transferase Gcnt1 deficient (*Gcnt1*^−/−^) mice^11^ display increased susceptibility to *Mtb* infection, mechanistically mediated by neutrophils and by Gcnt1 activity in stromal and hematopoietic compartments. Furthermore, we report for the first time altered expression of glycosyltransferases associated with sLe^x^ core-2 *O*-glycan biosynthetic pathway in whole blood of TB patients. Our findings support the modulation of *O*-glycosylation during *Mtb* infection, showing that alterations in this pathway compromise the ability of the host to deal with infection in the most competent way.

## Results

### Deficiency in Gcnt1 is associated with higher susceptibility to *Mtb* infection

**Fig. 1:**
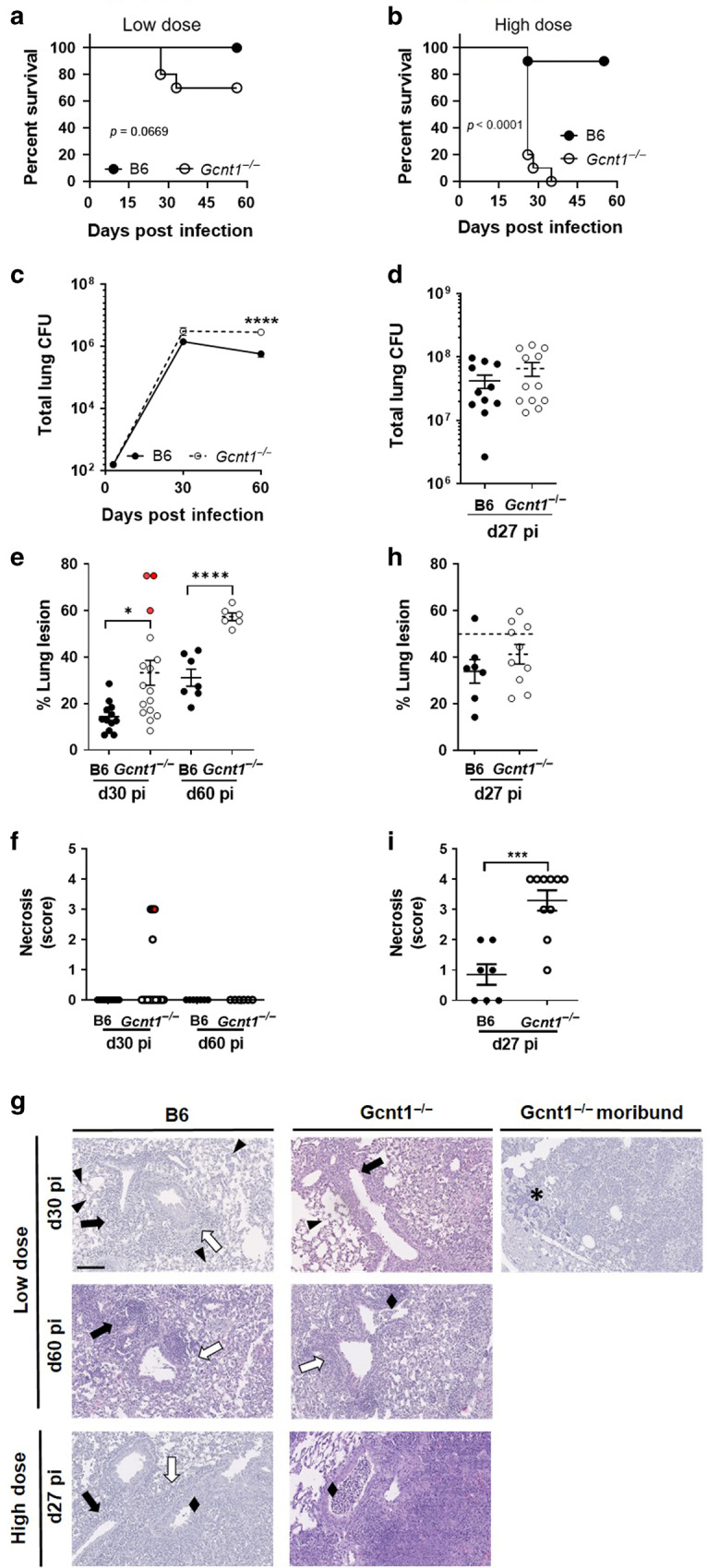
Deficiency in *Gcnt1* associates with increased susceptibility to *Mtb* infection. C57BL/6 (B6; black circles) or *Gcnt1*^−/−^ (open circles) mice were infected by aerosol with *Mtb* strain HN878 with a low (**a**, **c**, **e**–**g**) or high (**b**, **d**, **h**, **i**, **g**) dose of bacteria. **a**, **b** The weight of the animals was monitored to determine survival curves, that included 10–13 animals in two independent experiments. Statistical analysis was performed with a log-rank (Mantel-Cox) test for the Kaplan Meyer curve. **c**, **d** At the indicated timepoints post-infection, the lungs of infected mice were collected and the bacteria burden determined by CFU enumeration. **e**, **h** At the indicated timepoints post-infection, lung pathology defined as the percentage of infiltrate per lobe (Fig. S1A, B), was determined upon H&E staining and morphometric analysis of the right upper lobes of infected lungs. The histopathologic features of the infected lungs were assessed and a relative score attributed (Table 1); the score obtained for necrosis is plotted in **f** and **i**. The pictures in **g** are H&E staining of representative animals within each experimental group. Arrowheads point to intra-alveolar necrotic debris, black diamond point to bronchiolar debris, black arrows point to perivascular lymphocytes, white arrows point to peribronchiolar lymphocytes and asterisks to calcification sites. Scale bar corresponds to 100 µm. In **c** each dot represents the Mean ± SEM and in **d**–**h** each dot represents an individual mouse of 6–12 in at least two independent experiments. In **e**, **f** red dots represent moribund *Gcnt1*^−/−^ mice. Statistical analysis was performed using multiple *t*-test (**c**) or unpaired *t-*test (**d**–**i**). *p < 0.05; **p < 0.01; ***p < 0.01; ****p < 0.0001.

Studies with *Fut4*^*−/−*^ and *Fut7*^*−/−*^ hinted at a possible role for the host glycosylation machinery in TB.^19,20^ As Fut4 and Fut7 catalyse the terminal decoration of glycans, we reasoned that studying the impact of an earlier step of glycan biosynthesis would better reveal its biological effect during *Mtb* infection. Thus, we used mice lacking Gcnt1, a key enzyme for the biosynthesis of core-2 *O*-glycans.^11^ C57BL/6 and *Gcnt1*^−/−^ mice were infected via aerosol with *Mtb* strain HN878. Upon a low dose infection, 18.7% of *Gcnt1*^−/−^ mice succumbed around 30 days post-infection, whereas, as expected, all C57BL/6 mice survived (Fig. 1a). Strikingly, all *Gcnt1*^−/−^ mice infected with high doses of *Mtb* succumbed within 40 days, while 90% of C57BL/6 mice survived throughout the 60 days of the experiment (Fig. 1b). *Gcnt1*^−/−^ mice, which survived the low dose of infection, showed a higher lung bacterial burden than C57BL/6, particularly on day 60 post-infection (Fig. 1c). No significant differences in bacterial burdens of C57BL/6 or *Gcnt1*^−/−^ mice infected with high doses of *Mtb* for 27 days were observed (Fig. 1d), despite the different survival rates.

The percentage of lung lesion was quantified to measure lung pathology (Fig. S1A, B). As compared to C57BL/6, *Gcnt1*^−/−^ mice infected with low doses of *Mtb* showed increased lung pathology, which was mostly pronounced on day 60 post-infection with increased scores for perivascular and peribronchial lymphocytes (Table 1, Fig. 1e and Fig. S1C). The extent of lung lesion was particularly high for the *Gcnt1*^−/−^ mice, which succumbed to infection (Fig. 1e, Fig. S1D). Lung histopathologic analysis of the infected mice revealed an overall exacerbation of pathologic features in *Gcnt1*^−/−^ mice, with the moribund mice presenting severe inflammation, pronounced necrosis and calcification (Table 1, Fig. 1e–g). In infections with high doses of bacteria, a higher frequency of infected *Gcnt1*^−/−^ mice showed more than 50% of lung infiltrates as compared to C57BL/6, although globally no significant differences in relative lesion burden were noted (Fig. 1h). The histopathological features observed during infection with low doses of *Mtb* were recapitulated and exacerbated for those with high doses, with infected *Gcnt1*^-/-^ mice presenting extensive lung pathology, particularly extensive necrosis, and histological features of bronchopneumonia (Table 1, Fig. 1i, g and Fig. S1E).

**Table 1:** Lung histopathology analysis of Mtb-infected mice.

		Low dose					High dose	
		day 30		day 60		Moribund *Gcnt1*^−/−^ (3)	day 27	
	Score	B6 (12)	*Gcnt1*^−/−^ (13)	B6 (7)	*Gcnt1*^−/−^ (6)		B6 (7)	*Gcnt1*^−/−^ (10)
Perivascular lymphocytes	0							
	1	8.30						
	2	91.7	23.1	28.6		50.0	28.6	16.7
	3		76.9	71.4	100	50.0	71.4	83.3
Peribronchiolar lymphocytes	0							
	1	44.4	55.6	57.1	33.3		14.3	
	2	55.6	33.3	42.9	50	50.0	42.9	66.7
	3		11.1		16.7	50.0	42.9	33.3
Bronchiolar debris	0	70.0	66.7	42.9	50		14.3	
	1	30.0	22.2	57.1	33.3		57.1	10.0
	2		11.1		16.7	66.7	14.3	20.0
	3					33.3	14.3	70.0
Intra-alveolar necrotic debris	0							
	1	16.7	46.2	57.1	16.7	33.3		
	2	66.7	30.8	14.3	50	66.7	28.6	
	3	16.7	23.1	28.6	33.3		71.4	100
Necrosis	0	100	92.3	100	100		42.9	
	1						28.6	10.0
	2		7.70				28.6	10.0
	3					100		20.0
	4							60.0

Lung H&E sections of C57BL/6 (B6) or *Gcnt1*^−/−^ infected animals were assessed and scored for the indicated histopathological features. Indicated is the % of animals with a certain score within the indicated experimental group. Indicated in brackets is the number of animals analysed per group.

### Neutrophils drive the increased susceptibility of *Gcnt1*^−/−^ mice to *Mtb* infection

**Fig. 2:**
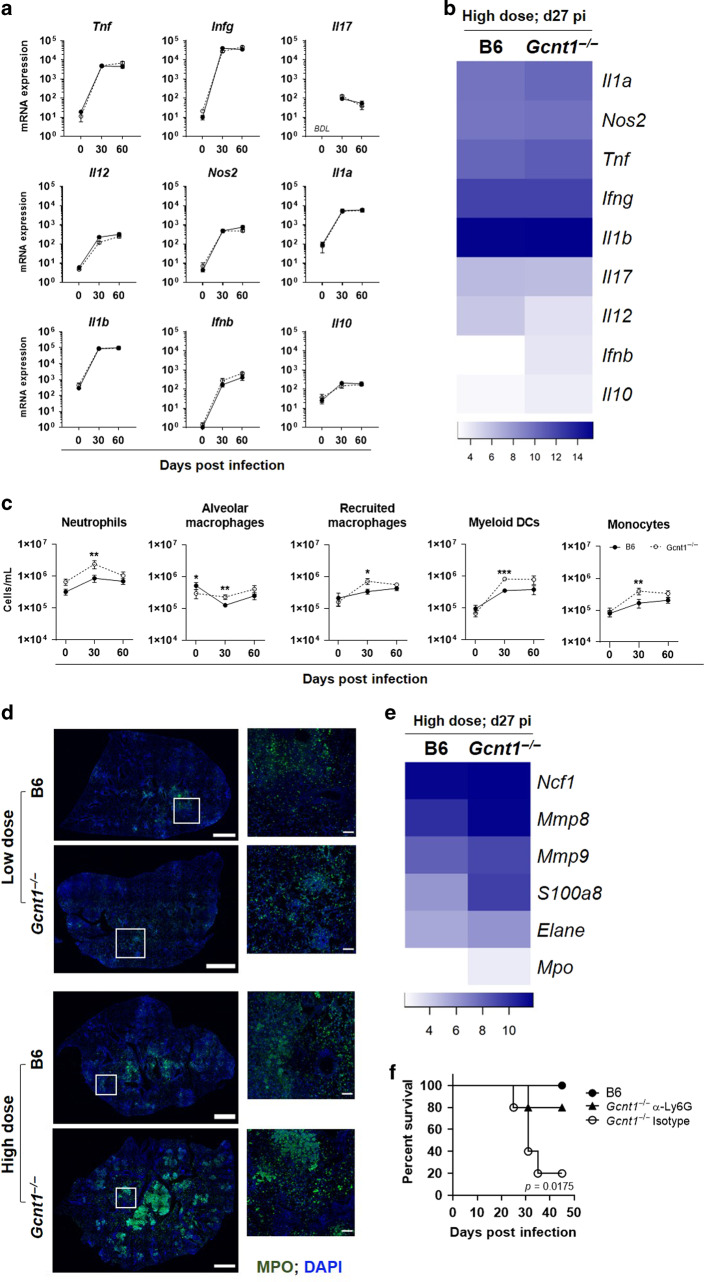
Exacerbated neutrophilia drives increased susceptibility of *Gcnt1*^−/−^ mice to *Mtb* infection. **a**–**e** At the indicated timepoints post-infection, the lungs of C57BL/6 (B6; black circles) or *Gcnt1*^−/−^ (open circles) mice infected by aerosol with a low or high dose of *Mtb* strain HN878 were harvested and a cellular suspension prepared. **a**, **b**, **e** The relative expression of the indicated genes was determined by real-time PCR. **c** Percentages of the indicated immune cell populations were determined by flow cytometry. The gating strategy is shown in Fig. S2A. **d** Representative images of MPO staining (green) in lung sections of B6 and *Gcnt1*^−/−^ mice infected with low or high doses of *Mtb*. Sections were counterstained with DAPI (blue). Scale bars correspond to 1 mm (left panels) and 100μm (right panels). In **a**, **c** each dot represents the Mean ± SEM for 10 animals in two independent experiments. Non-infected animals (day 0) were used as controls. **b**, **e** Represented are heatmaps of log2 relative expression of the indicated genes in lung samples of B6 and *Gcnt1*^−/−^ mice 27 days after high dose *Mtb* infection. **f** C57BL/6 (B6, black circles) or *Gcnt1*^−/−^ mice were infected by aerosol with a high dose of *Mtb* strain HN878. On day 18 post-infection the *Gcnt1*^−/−^ mice were treated with the neutrophil-depleting anti-Ly6G mAb (black triangles) or an isotype control (open circles). The weight of the animals (5 per group) was monitored to determine survival curves. Statistical analysis was performed per time point with unpaired two-tailed Mann-Whitney test (**a**, **c**) or with log-rank (Mantel-Cox) test for the Kaplan Meyer curve (**f**). * refer to statistic differences between C57BL/6 or *Gcnt1*^−/−^ mice. *p < 0.05; **p < 0.01; ***p < 0.001.

To investigate the mechanisms underlying the increased susceptibility of *Gcnt1*^−/−^ mice to *Mtb*, we compared the dynamics of the immune response in C57BL/6 or *Gcnt1*^−/−^ mice during infection with low or high doses of bacteria. No significant differences were noted in the dynamics or levels of expression of genes encoding key inflammatory mediators for the immune response against *Mtb* infection with low doses (Fig. 2a). Similarly, no differences were found between non-infected *Gcnt1*^−/−^ or C57BL/6 mice (Fig. 2a). Upon infection with high doses of *Mtb*, we noted an overall higher expression of cytokines in *Gcnt1*^*−/−*^ animals, as compared to C57BL/6 ones (Fig. 2b). In all, the lung pathology observed in infected *Gcnt1*^−/−^ mice does not seem to result from an uncontrolled cytokine storm.

The dynamics of recruitment of myeloid and lymphoid cells to the lung was identical in *Gcnt1*^−/−^ and C57BL/6 mice infected with a low dose of bacteria, but 30 days after infection all tested immune cell populations were significantly higher in the lung of *Gcnt1*^−/−^ as compared to C57BL/6 mice (Fig. 2c and Fig. S2A, B). Moreover, increased numbers of lung neutrophils were detected in *Gcnt1*^−/−^ mice, independently of the infection (Fig. 2c). Because lung infiltrating neutrophils have been associated with increased bacterial burdens, pathology and TB exacerbation,^1–4^ we assessed the contribution of neutrophils to the increased susceptibility of *Gcnt1*^−/−^. For that, we investigated the distribution of neutrophils in the lungs of C57BL/6 or *Gcnt1*^−/−^ mice infected with low or high doses of bacteria, through detection of myeloperoxidase (MPO) expression by immunostaining. Although neutrophils were present in both mouse strains upon infection, these cells accumulated in the case of infected *Gcnt1*^−/−^ mice (Fig. 2d), in line with the cytometry data. An accumulation of neutrophils in the areas of lesion was particularly observed in infections with high doses of *Mtb*, being more exacerbated in *Gcnt1*^−/−^ mice (Fig. 2d). A similar accumulation was found in the necrotic lesions of moribund *Gcnt1*^−/−^ mice infected with low doses of bacteria (Fig. S2C). Moreover, and in line with the higher presence of neutrophils in the lungs of *Gcnt1*^−/−^ mice infected with high doses of *Mtb*, the expression levels of genes previously associated with a neutrophil-dominated transcriptional module^21^ were increased in these animals, as compared to C57BL/6 ones (Fig. 2e). We next treated *Mtb*-infected *Gcnt1*^−/−^ mice with a neutrophil-depleting antibody^22^ or with an isotype control (Fig. S2D). To assess mouse survival as the readout of this experiment, a high dose of infection was performed. Whereas 80% (*n* = 4) of isotype control-treated *Gcnt1*^−/−^ mice died between 25–35 days post-infection, only 20% (*n* = 1) of those treated with the Ly6G depleting antibody did not survive and all C57BL/6 mice survived infection (Fig. 2f). Therefore, depleting neutrophils largely rescued the increased susceptibility of *Gcnt1*^−/−^ mice to *Mtb* infection. Finally, we compared the response of neutrophils isolated from mice of either genetic background to in vitro *Mtb* infection. Although no statistically significant differences were found, we noted an overall enhanced cytokine expression by *Gcnt1*^−/−^ neutrophils upon *Mtb* infection (Fig. S2E), but similar production of reactive oxygen and nitrite species (Fig. S2F). In all, these findings suggest that *Gcnt1* abrogation did not alter the basic functional response of neutrophils to *Mtb*.

### Gcnt1 activity modulates granulopoiesis and lung neutrophil recruitment

**Fig. 3:**
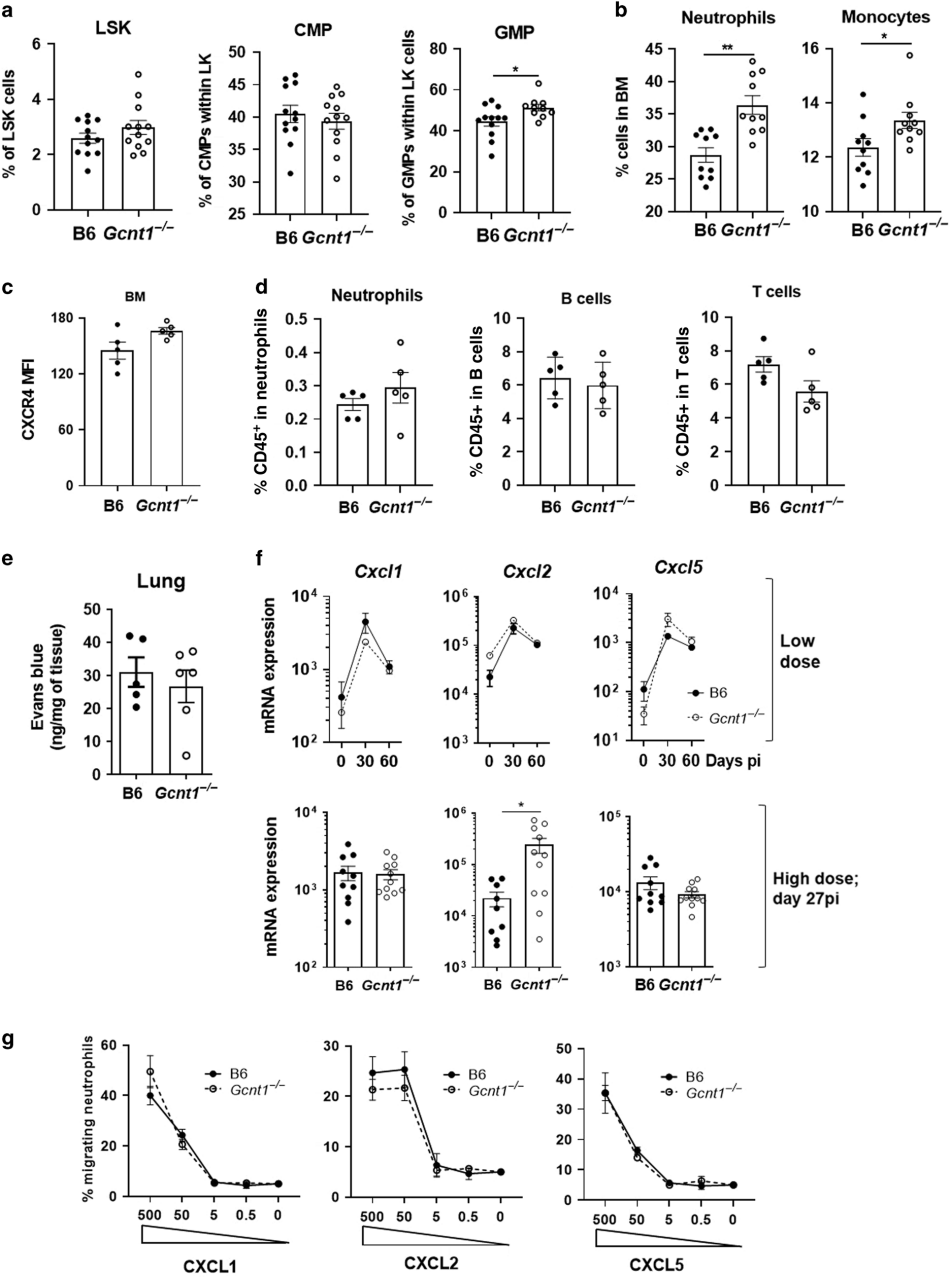
Gcnt1 modulates granulopoiesis and CXCL2 expression. The BM of non-infected C57BL/6 (B6; black circles) or *Gcnt1*^−/−^ (open circles) mice was harvested and the frequency of **a** LSK, CMP and GMP populations; **b** neutrophils and monocytes or (**c**) neutrophils expressing CXCR4 determined by flow cytometry. All gating strategies are shown in Fig. S3. **d** Egress of immune cell populations from the BM of in C57BL/B6 (B6; black circles) or *Gcnt1*^−/−^ (open circles) measured as the percentage of CD45^+^ BM sinusoidal cells upon intravenous injection of CD45-PE. **e** Permeability of the lung vasculature in C57BL/B6 (B6; black circles) or *Gcnt1*^−/−^ (open circles) measured through quantification of the amount of Evans Blue extravasated into the tissue upon intravenous injection. **f** The expression of *Cxcl1*, *Cxcl2* and *Cxcl5* in the lungs of mice infected with low or high doses of *Mtb* was determined by real-time PCR, for the indicated timepoints post-infection. **g** Analysis of neutrophils migration through 5 μm transwells towards different concentrations of recombinant CXCL1, CXCL2 and CXCL5. Mean ± SEM of culture triplicates are presented. In **a**, **f** each dot represents a mouse and the Mean±SEM for 6–15 animals in at least two independent experiments are plotted. Statistical analysis was performed with Student’s *t*-test (**a–f**) or unpaired two-tailed Mann-Whitney test for each time point (**g**). * refer to statistic differences between B6 or *Gcnt1*^−/−^ mice. *p < 0.05; **p < 0.01; ***p < 0.01; ****p < 0.0001.

We next investigated the causes underlying *Gcnt1*^−/−^ mice neutrophilia. Blood neutrophilia was reported for healthy *Gcnt1*^−/−^ mice.^11^ We here confirmed this finding and further showed its maintenance throughout infection (Fig. S3A,B). To test whether lack of Gcnt1 may favour granulopoiesis, we analysed the frequency of Lin^−^Sca1^+^cKit^+^ cells (LSK), common myeloid precursors (CMP), granulocytic-monocytic precursors (GMP) and mature cell populations in the bone marrow (BM) of C57BL/6 or *Gcnt1*^−/−^ mice (Fig. S3C,D). No differences were seen in the frequencies of LSK and CMP progenitor cell populations, but an increased frequency of GMPs was noted (Fig. 3a). In line with this increase in GMP, the frequencies of neutrophils and monocytes were higher in the BM of *Gcnt1*^*−/−*^ mice (Fig. 3b). As for lymphoid populations, a decreased frequency of B cells and a similar frequency of T cells were observed in *Gcnt1*^*−/−*^ BM, as compared with C57BL/6 ones (Fig. S3E). Thus, the BM and blood neutrophilia observed in *Gcnt1*^−/−^ mice may at least in part result from enhanced BM granulopoiesis.

We next investigated the impact of Gcnt1 deficiency on the mobilization of the neutrophils between BM and blood. As CXCR4 is a key regulator of neutrophil retention versus egress from the BM,^23,24^ we compared the expression of this receptor in BM neutrophils of each mouse strain. The levels of CXCR4 expressed in BM neutrophils were comparable in C57BL/6 and *Gcnt1*^−/−^ mice (Fig. 3c). Furthermore, to investigate for differences in the egress of neutrophils from the BM in *Gcnt1*^−/−^ mice, we intravenously injected an antibody directed to CD45 and assessed the percentage of CD45^+^ BM sinusoidal cells in either mouse strain. No differences were found in the frequency of CD45 labelled neutrophils, B cells and T cells when comparing the two mouse strains (Fig. 3d). Together, these data suggest that *Gcnt1* deficiency does not alter the hematopoietic cell retention in the BM or egress from the BM to the blood.

We then sought to understand the cause for increased lung neutrophil frequencies in *Gcnt1*^−/−^ mice. We started by testing the permeability of the lung vasculature by measuring the influx of Evans blue injected intravenously. Abrogation of *Gcnt1* did not impact the  diffusion of dye into the lung (Fig. 3e), indicating a similar permeability of the lung vasculature in *Gcnt1*^−^ and C57BL/6 mice. Next, we questioned whether the lung neutrophilia of *Gcnt1*^−/−^ mice depended on altered neutrophil chemoattraction. For this, we measured the expression levels of lung *Cxcl1*, *Cxcl2* and *Cxcl5* during infection with low or high doses of *Mtb* (Fig. 3f). Although the transcription of these chemokines in lungs of C57BL/6 or *Gcnt1*^−/−^ mice was similar upon a low dose of infection, the expression of *Cxcl2* was higher in *Gcnt1*^*−/−*^ deficient mice than in C57BL/6 upon a high dose of infection (Fig. 3f). Finally, we tested the ability of neutrophils from either genetic background to migrate in response to each of these chemokines, using an in vitro trans-well system. Independently of the expression of *Gcnt1*, the migration of neutrophils towards CXCL1, CXCL2 or CXCL5 was dose dependent and similar (Fig. 3g). Collectively, these findings support a higher availability of neutrophils in the absence of Gcnt1, accompanied by an increased recruitment of neutrophils to the lung of *Gcnt1*^−/−^-infected mice, possibly mediated by the higher expression of CXCL2.

### Increased susceptibility to *Mtb* infection requires Gcnt1 deficiency in both hematopoietic and non-hematopoietic compartments

**Fig. 4:**
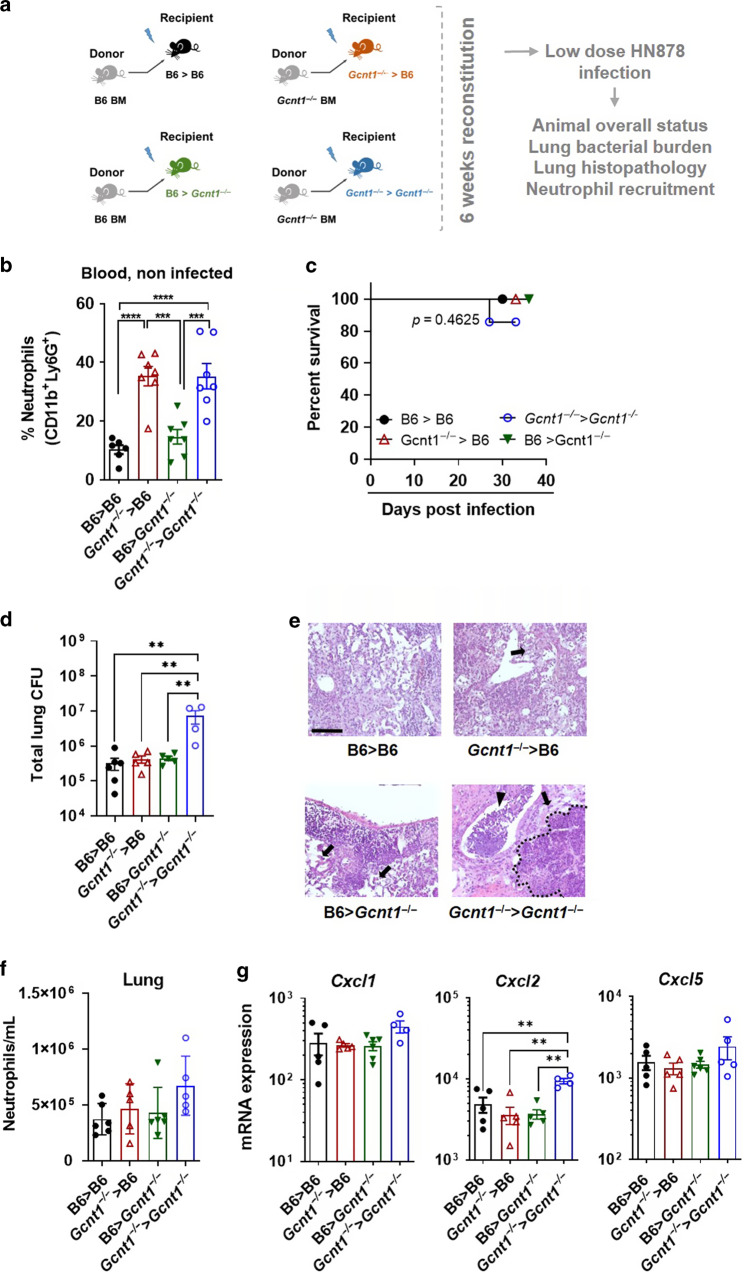
Blood neutrophilia of *Gcnt1*^−/−^ mice is promoted by deficiency of this enzyme in hematopoietic cells, but increased susceptibility to Mtb infection also requires the non-hematopoietic compartment. **a** Schematic representation of the BM transplantation model used and the experimental groups included. **b** The frequency of neutrophils in the blood of non-infected chimeric mice was determined by flow cytometry, following the gating strategy shown in Fig. S3. **c** Mice in the different chimeric groups were infected with a low dose of *Mtb* strain HN878 and the weight of the animals monitored to determine survival curves. **d** On day 30 post-infection, the bacterial burden in the lungs of the infected mice was determined by CFU enumeration. **e** H&E staining of one representative animal of each experimental group is represented. Black arrows point to edema, black arrowhead spot bronchopneumonia, and dashed black line limits the necrotic areas. Scale bar corresponds to 100 µm. **f** The number of neutrophils present in infected lungs was determined on day 30 post-infection, by flow cytometry. **g** The expression of *Cxcl1*, *Cxcl2* and *Cxcl5* in the lungs of infected mice was determined by real-time PCR, as above. **b**, **d**, **f**, **g** Represented is Mean±SEM, and each symbol represents one mouse. Statistical analysis was performed with a one-way ANOVA using Tukey’s test for multiple comparisons (**b**, **d**, **f**, **g**) or with log-rank (Mantel-Cox) test for the Kaplan Meyer curve (**c**). * refer to statistic differences between the indicated chimeric groups. **p < 0.01; ***p < 0.001; ****p < 0.0001.

To investigate if the susceptibility of *Gcnt1*^−/−^ mice to *Mtb* infection was fully dependent on the hematopoietic compartment, we resorted to a mouse BM transplantation model (Fig. 4a and Fig. S4A). When comparing all experimental groups, we observed that both the *Gcnt1*^−/−^→C57BL/6 and the *Gcnt1*^−/−^→*Gcnt1*^−/−^ chimeric animals presented significantly higher neutrophils in circulation upon reconstitution and before infection (Fig. 4b), suggesting that hematopoietic deficiency of Gcnt1 is sufficient to promote neutrophilia. Chimeric mice were then infected with a low dose of *Mtb*. Mice in the *Gcnt1*^−/−^→*Gcnt1*^−/−^ group presented a different survival curve compared to all other groups, where one animal (representing 14.3%) succumbed to infection after 24 days (Fig. 4c), and also higher lung bacterial burdens 30 days post-infection than any other group (Fig. 4d), paralleling the increased susceptibility to infection of *Gcnt1*^−/−^ mice. Histopathological analysis of the infected chimeric animals showed that although *Gcnt1*^−/−^→C57BL/6 mice presented edema and larger necrotic areas when compared to the C57BL/6→C57BL/6 group, *Gcnt1*^−/−^→*Gcnt1*^−/−^ animals presented the most exuberant pathology with severe inflammation, edema, vast necrosis and bronchopneumonia (Fig. 4e and Fig. S4B). Lung flow cytometry analysis of the infected chimeric mice revealed an overall higher frequency of neutrophils in the lungs of *Gcnt1*^−/−^→*Gcnt1*^−/−^ chimeric mice (Fig. 4f) and similar frequencies of all other cell populations (Fig. S4C). Interestingly, in line with what we had observed in *Gcnt1*^−/−^ infected mice, the expression of *Cxcl2* upon infection was exclusively increased in *Gcnt1*^−/−^→*Gcnt1*^−/−^ chimeric mice (Fig. 4g).

Thus, although *Gcnt1* deficiency in the hematopoietic compartment is sufficient to deregulate blood neutrophil frequencies in homeostasis, for that to translate into different susceptibilities to *Mtb* infection, deficiency of *Gcnt1* in the stromal compartment is also required.

### *Mtb* infection modulates the host glycosylation machinery at the gene transcription level

**Fig. 5:**
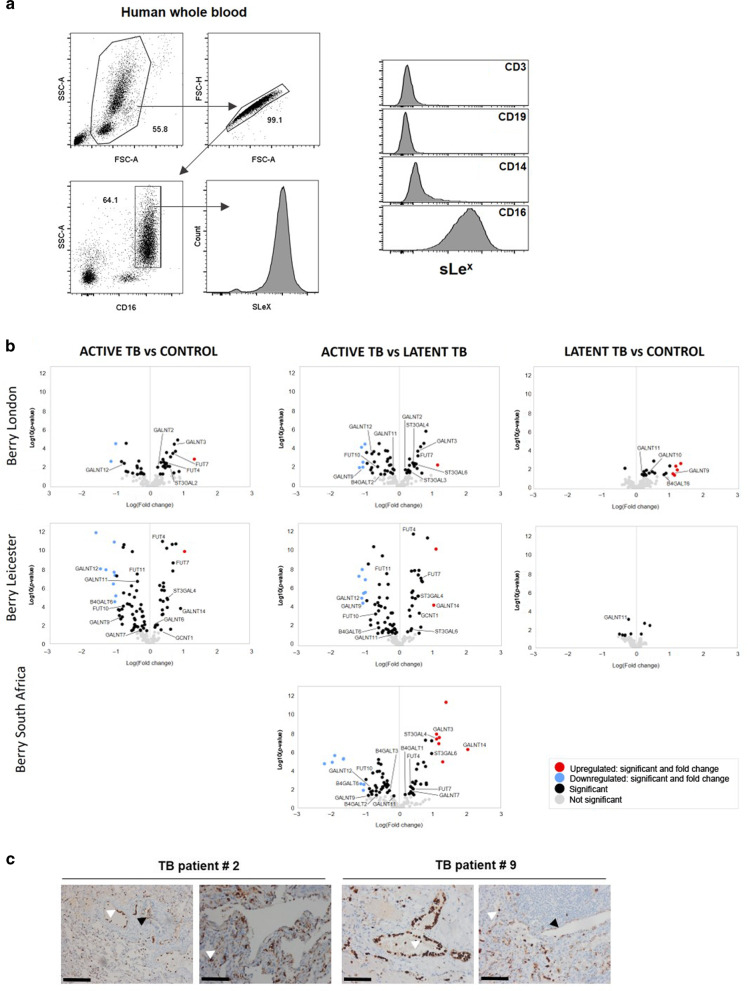
*Mtb* infection impacts the expression of several glycosyltransferase-encoding genes in humans. **a** Neutrophils are the main cells expressing sLe^x^ in human peripheral blood. Vein blood was collected and stained for CD3, CD14, CD16, CD19 and sLe^x^ by flow cytometry. Data shown represent one donor out of 25 analysed. **b** Volcano plots displaying the comparisons between Active TB vs Control, Active TB vs Latent TB and Latent TB vs Control regarding the differential expression of annotated coding genes of glycosyltransferases (obtained from Glyco-Enzyme Repository) on the Berry London, Berry Leicester (progressor) and Berry South Africa datasets.^26^ Labelled genes encode for glycosyltransferases associated with the sLe^x^ pathway. Dot color indicates if the corresponding gene is upregulated (red, log-fold-change >1 and *p*-value < 0.05), downregulated (blue, log-fold-change < -1 and *p*-value < 0.05), significant (black, log-fold-change >−1 to <1 and *p*-value < 0.05) and not significant (grey). **c** Lung sections of TB patients who underwent therapeutic surgery were stained for sLe^x^ as above. Black and white arrowheads point to positive epithelial or immune cells, respectively. Scale bars correspond to 100 µm.

Our findings highlight the importance of a competent core 2 *O*-glycan pathway in regulating neutrophils and immune pathology during TB. Core-2 *O*-glycans are major carriers of terminal Lewis antigens and, among these, sLe^x^ is described to decorate the surface of human neutrophils (Fig. 5a). Because a neutrophil-driven signature was observed in the whole blood of TB patients,^25^ and since our data link Gcnt1 activity with TB susceptibility through a mechanism involving neutrophils, we next interrogated available human whole blood TB RNA-Seq datasets^26^ for transcriptional alterations in glycosyltransferase-encoding genes (Table S1). Several glycosyltransferase-encoding genes were significantly up- or downregulated in active TB patients as compared to control or latently infected individuals (Fig. 5b). In particular, significant alterations in genes encoding enzymes of the sLe^x^ biosynthetic pathway were detected (Fig. 5b). Importantly, a much lower level of variation was observed when latent and controls were compared (Fig. 5b), thus showing that TB disease impacts the glycosyltransferase transcriptomic signature. Lastly, we examined lungs of TB patients for the presence of sLe^x^ antigens. TB patients (Table 2) were enrolled in a study in Georgia and underwent lung surgery as a therapeutic option, after antibiotherapy failure.^27^ Independently of the patient’s gender and *Mtb* drug susceptibility profile, lung sLe^x^ expression was detected in epithelial and immune cells (Fig. 5c and Fig. S5).

**Table 2:** Overall characteristics of the participants originating lung samples.

Patient	Gender	Lesion localization^a^	Lesion size (mm)	TB symptoms	Drug sensitivity profile^b^
1	Male	RUL	21, 30	Yes	DS
2	Male	RUL	35	Yes	DS
3	Female	LLL	30	No	MDR
4	Female	LLL	30	No	DS
5	Female	RUL	30	No	DS
6	Female	RLL	42	No	MDR
7	Male	RUL	30, 21	No	MDR
8	Female	LUL	40	No	DS
9	Male	LUL	38	No	MDR
10	Male	LUL	29	No	MDR

All patients were antibiotic-treated before surgery. Highlighted in grey are the patients represented in Fig. 5.

*RUL* right upper lobe, *LLL* left lower lobe, *RLL* right lower lobe, *LUL* left upper lobe, *DS* drug sensitive, *MDR* multi-drug resistant.

Altogether, we provide, for the first time, evidence for the modulation of core 2-sLe^x^ antigens during human TB, through the transcriptional regulation of various glycosyltransferase-encoding genes.

## Discussion

Despite the growing significance of glycosylation in immunity,^5,28^ its impact in TB is poorly understood. To address this hitherto unexplored question, we studied the outcome of *Mtb* infection in mice lacking Gcnt1, a key glycosyltransferase controlling core 2 *O*-glycans biosynthesis. Gcnt1 deficiency impacted bacterial burdens, lung pathology and mouse survival. It also associated with exacerbated BM, blood and lung neutrophilia, already present in naïve animals. In keeping with the immune pathological role of neutrophils in TB,^1–4^ the accumulation of these cells was strongly associated with lung necrotic lesions and it was markedly increased in *Gcnt1*^−/−^ infected mice. Further implicating neutrophilia with susceptibility of *Gcnt1*^−/−^ mice to *Mtb* infection, depleting neutrophils from *Gcnt1*^−/−^ mice largely rescued their susceptible phenotype. Interestingly, *Fut4*^−/−^ mice present normal neutrophil counts^29^ and no phenotype upon *Mtb* infection,^19,20^ whereas *Fut7*^−/−^ mice exhibit blood neutrophilia^30^ and increased susceptibility to *Mtb* infection.^19,20^ Moreover, our preliminary data show that *St3gal4*^−/−^ mice present normal neutrophil counts in homeostasis and respond to *Mtb* infection similar to C57BL/6 mice.

Blood neutrophilia has been described in *Gcnt1*^−/−^ mice,^11^ but the underlying mechanisms remain elusive. Here, we show that hematopoietic abrogation of Gcnt1 was sufficient to cause blood neutrophilia, which may result from enhanced BM granulopoiesis. We have excluded other possible regulatory mechanisms, including a direct modulation of CXCR4 receptor levels in *Gcnt1*^*−/−*^ neutrophils or a differential egress of immune cells from the BM to the blood. We also report an increase in lung neutrophil counts in *Gcnt1*^−/−^ mice, which may seem counterintuitive, as absence of Gcnt1 associates with a partial deficiency of selectin ligands.^11^ However, the recruitment of neutrophils to the lung may follow an atypical selectin-independent cascade,^9,31^ as shown during *Streptococcus pneumonia* infection.^32^ Also, Gcnt1 activity has been demonstrated to be dispensable for lung neutrophil recruitment upon LPS stimulation.^33^ We show that lung neutrophilia in *Gcnt1*^−/−^ mice involved both hematopoietic and stromal compartments. Whereas the contribution of the hematopoietic deficiency may be related to the increased number of available circulating neutrophils, that of the stroma does not relate with alterations in the permeability of the lung vasculature and may result from an upregulated *Cxcl2* expression. Interestingly, it does not rely on differential expression of *Cxcl5*, a chemokine produced by epithelial cells that drives neutrophil recruitment, contributing to more severe forms of TB.^34^ In the same context, *Gcnt1*^−/−^ neutrophils showed migratory profiles towards CXCL1, CXCL2 and CXCL5 similar to those of C57BL/6 neutrophils.

Neutrophils are widely described as detrimental in TB,^1–4^ and an interferon-inducible neutrophil-driven blood transcriptional signature was defined in human TB.^25^ Interestingly, human neutrophils are decorated with sialylated antigens, most notably sLe^x^, which biosynthetic pathway involves Gcnt1 enzymatic activity.^7^ We found alterations in the expression of several glycosyltransferases in whole blood of active TB patients as compared to controls. Furthermore, we detected sLe^x^ immunostaining in lung sections of TB patients. Of note, previous reports showed that the urinary levels of sialic acid, the terminal glycan of sLe^x^ structure, discriminate patients with active TB from healthy controls or patients with non-tuberculous pulmonary diseases.^35^ Also, the regulation of the gene encoding CMAS, the enzyme that provides the substrate for the addition of sialic acid, specifically changed with infection with *Mtb*.^36^ Thus, modulation of sialylated antigens seems to occur in TB, as described in several pathologies, such as other infectious diseases and cancer.^6,16,17,37^

Collectively, this study further strengthens the role of glycosylation during immune responses and reveals that regulation of neutrophil homeostasis by host glycosyltranferases impacts TB outcome, a disease connected with high mortality and morbidity. Our findings raise awareness for the possible detrimental impact of targeting glycan dependent interactions, as envisaged for example in anticancer therapies,^38^ in the context of TB. Additionally, a better understanding of glycans and their functional roles during chronic infection, provides increasing opportunities for discovery and therapeutic intervention in settings like TB.

## Material and methods

### Ethics statement

The lung samples study was reviewed and approved by the Ethics Committees of the National Center for Tuberculosis and Lung Diseases of Georgia (IRB00007705-NCTLD-Georgia#1, IORG0006411) and of the Germans Trias I Pujol Hospital (EC:PI-16–171). The blood samples study was reviewed and approved by the Portuguese *Comissão de Ética para a Saúde da ARS Norte* (#T792). All participants signed informed consents, and all data were anonymized.

Animal experiments followed the 2010/63/EU Directive, and were approved by the i3S Animal Ethics Committee and the Portuguese National Authority for Animal Health (#014811/2016-07-13) or by the Animal Experimentation Ethics Committee of the Hospital Universitari Germans Trias i Pujol (#B9900005) and the Dept d’Agricultura, Ramaderia, Pesca, Alimentació i Medi Natural of the Catalan Government.

### Animals

C57BL/6 wild-type or *Gcnt1*^*−/−*^ mice were bred and housed at i3S and infected under ABSL3 conditions. The *Gcnt1*^*−/−*^ mouse was obtained from the Consortium for Functional Glycomics.^11^ Mice were euthanized with isoflurane or CO_2_ inhalation with efforts to minimize suffering. Food and water were *ad libitum*.

### Bacteria growth and quantification

*Mtb* strain HN878 was grown and stored as before.^39^ Viable bacteria was determined by serial dilution and colony forming unit (CFU) enumeration after 21–28 days of incubation at 37 °C in 7H11 agar plates. Bacterial quantification in infected lungs followed a similar protocol.

### Experimental infection and animal monitoring

C57BL/6 and *Gcnt1*^−/−^ (8–12 week old) mice were aerosol-infected with *Mtb* strain HN878 using an inhalation exposure system (Glas-Col), as published.^39,40^ Low and high dose infections delivered <200 or >350 bacteria to the lung, respectively, as determined 3 days post-infection. Infected mice were weighted every week or every two days, and humanely euthanized if 20% of their weight or responsiveness to physical stimulation were lost. Whenever possible, the lungs of moribund animals were harvested for histology assessment.

### Organ processing

Lungs were aseptically excised and processed as before.^39,40^ Single-cell suspensions were used for bacterial burden determination, flow cytometry and RNA analysis.

### Tissue samples, histology and morphometric analysis

The human lung samples (Table 2) are from the collection obtained within the SH-TBL project, led by the Experimental Tuberculosis Unit (UTE) and conducted in collaboration with the NCTLD; and registered at the ClinicalTrials.gov database under code NCT02715271.

Mouse lungs were fixed in 10% buffered-formalin and paraffin-embedded. Serial sections of 3 µm-thickness were haematoxylin-eosin (H&E) or Ziehl-Neelsen stained or used for immunofluorescence (MPO) analysis.^39^ Morphometric quantification of lung histology images is detailed in Fig. S1. Pathological scoring analysis of H&E-stained histological sections was performed by a veterinary pathologist blinded to the treatment groups. The histopathological features were scored using the following scale: 0 = absent, 1 = focal 2 = multiple, 3 = severe, 4 = severe and extensive. The % of animals with a certain score within the indicated experimental group was calculated for group comparison.

### MPO immunostaining

Lung sections were stained with a goat anti-mouse antibody against MPO (AF3667, R&D systems; 1:40) overnight at 4 °C followed by a secondary donkey anti-goat IgG antibody conjugated with Alexa Fluor 568 (A11057, Invitrogen; 1:1000), and DAPI (Biolegend; 1:1000) for 2 h in the dark at RT. Glass coverslips were mounted onto the slides using Vectashield (Sigma). Slides were acquired using the IN Cell Analyzer 2000 (GE Healthcare) and analysed using IN Cell Developer software and ImageJ.^41,42^

### RNA extraction, cDNA synthesis and real-time PCR analysis

Total RNA was extracted from mouse lungs or in-vitro-infected neutrophils, using TRI-Reagent (Sigma), converted to cDNA (NE Biolabs), and subjected to real-time PCR using SYBR green (Applied Biosystems) and specific oligonucleotides (Table S2). Melting and standard curves and RQ values were determined for each gene. Ubiquitin was used as a reference gene for normalization of target gene abundance.

### Flow cytometry

Human or mouse blood, mouse BM and lung were stained for surface antigens (30 min; 4 °C) and fixed for 20 min in 2% paraformaldehyde-PBS after erythrocyte lysis. For the analysis of BM precursors, the mature lineage (Lin) was depleted (Miltenyi Biotec magnetic cell separation system), prior to Lin^neg^ staining with specific antibodies for progenitor markers. Dead cells were always excluded using a viability dye. Cells were acquired on a BD FACS Canto II. Data were analyzed using FlowJo version 10.1.r7. Gating strategies are in Fig. S2 and S3 and the antibodies used in Table S3.

### Neutrophil depletion

In vivo depletion of neutrophils from infected *Gcnt1*^-/-^ mice was as described:^22^ 0.2 mg anti-Ly6G mAb (clone 1A8; BioXCell) or isotype control (clone GL117; R&D Systems) were administered i.p. from day 18 post-infection, every other day for 20 days. To control for the protocol, neutrophils in the lungs of mice treated with anti-Ly6G mAb or isotype control were detected at the end of the experiment by MPO staining (Fig. S2D).

### Analysis of BM egress

Mice were intravenously injected with 0.6 μg of anti-mouse CD45 conjugated with Phycoerythrin (PE) (30-F11; Biolegend) in PBS, 2 min prior to euthanasia, to label BM sinusoidal cells.^43^ Blood and BM cells were collected and analysed by flow cytometry.

### Analysis of lung vasculature permeability

Vasculature permeability was assessed using Evans Blue dye as described previously.^44,45^ Mice were intravenously injected with Evans Blue (1 mg per 30 g mouse, prepared in PBS). Thirty minutes after injection mice were sacrificed and lungs collected. After weight determination, samples were incubated in formamide for ~36 h at 55 °C. The formamide solution was recovered and absorbance was measured at 620 nm and 740 nm to determine the amount of Evans Blue present in the tissue. The presence of heme groups was corrected with the formula: A_620_(corrected) = A_620_ − (1.426 × A_740_ + 0.030).

### In vitro infection of neutrophils and chemoattraction assays

For BM neutrophils isolation, BM cells were stained with anti-mouse Ly6G (1A8; Biolegend) followed by purification using anti-biotin magnetic beads and LS columns of MACS cell separation system (both Miltenyi Biotec). For in vitro infection, 10^5^ neutrophils were seeded per well and infected with *Mtb* HN878 at MOI 2 or 10. After 3 h of infection, cells were recovered for RNA analysis or reactive oxygen species detection with DHE and DHR probes. Cells were incubated with DHE or DHR at 37 °C for 10 or 30 min, respectively. Samples were then acquired on a BD FACS Canto II to detect red-fluorescence (DHE) or green fluorescence (DHR) due to probes oxidization. For the chemotaxis assay, 10^5^ neutrophils were seeded on the upper chamber of the 5 μm-pore transwells (96 well plate; Corning) and allowed to migrate for 3 h towards the lower chamber containing media with CXCL1, CXCL2 and CXCL5 (all from Peprotech) at 500, 50, 5 and 0.5 ng/ml or without cytokines. As control for the amount of cells seeded, cells were also seeded in the lower chamber. Cells were then recovered and stained with SAV PE-Cy7 (Biolegend). To quantify the number of migrated cells, counting beads (Biolegend) were added to the cells prior to acquisition in a BD FACS Canto II. All cytometry data were analysed using FlowJo version 10.1.r7.

### Generation of BM chimeric mice

Recipient mice were irradiated with doses of 750 rad and reconstituted with 5 × 10^6^ CD3-TCRβ-TCRγ/δ-depleted BM cells. Bactrim (80 mg sulfametoxadol and 16 mg trimetoprim/250 mL of water, for an estimated dose of 50 mg antibiotic/Kg/day) was administered in drinking water for the first 3 weeks post-reconstitution. A control group, in which BM cells from CD45.1-C57BL/6 mice were transferred into irradiated CD45.2-*Gcnt1*^−/−^ mice, was included to monitor the efficiency of chimerism, 6 weeks post-transplantation (Supplemental Fig. 3A). Chimeras were infected at this stage.

### RNA-seq data analysis

Raw paired-end RNA-seq data from whole-blood cohorts Berry London, Berry South Africa and Berry Leicester progressor^26^ (GSE107995) were processed separately. The reads were quality controlled through FastQC [v0.11.7] (Babraham Bioinformatics) and MultiQC.^46^ Filtering below 20 quality score and below 50 base-pairs, and adapters exclusion was performed using Trimmomatic [v0.38].^47^ Filtered reads were aligned to the human reference genome GRCh38 using HISAT2 [v2.1.0].^48^ StringTie [v1.3.4] was used for the assembly and raw counts matrix generated by prepDE.py.^49^ Differentially expressed genes were analysed in R [v3.5.1] with Bioconductor packages edgeR^50,51^ and limma.^52^ Genes with less than 15 raw counts in all samples were excluded. 202 genes encoding glycosyltransferases was extracted from the Glyco-Enzyme Repository (http://glycoenzymes.ccrc.uga.edu/). Differentially expressed genes were determined through linear model fitting. Empirical Bayes moderated *t*-statistics test was performed and genes with p-value < 0.05 and log2 fold-change < −1 or >1 were considered significant.

### Immunohistochemistry and image analysis

Human lung sections were immunolabeled for sLe^x^ as described,^53^ with an antibody directed against CD15s (BD Pharmingen; CSLEX1) and ultraView DAB (Ventana Medical Systems).^54^

### Statistical analysis

Data were analysed using GraphPad Prism (version 8.1.0). Student’s *t*-test was used to compare two groups and one-way ANOVA for more than two groups, with post-tests as in Figure legends. Data were checked for normality and log normality. Survival curves were analysed using Log-rank (Mantel-Cox) test. Significant differences are as follows: *p ≤ 0.05; **p ≤ 0.01; ***p ≤ 0.001 and ****p ≤ 0.0001.

## Supplementary information

Supplementary Information

## References

[1] Dorhoi A, Kaufmann SH (2016). Pathology and immune reactivity: understanding multidimensionality in pulmonary tuberculosis. Semin. Immunopathol..

[2] Eruslanov EB (2005). Neutrophil responses to Mycobacterium tuberculosis infection in genetically susceptible and resistant mice. Infect. Immun..

[3] Marzo E (2014). Damaging role of neutrophilic infiltration in a mouse model of progressive tuberculosis. Tuberculosis (Edinb.).

[4] Lowe DM (2013). Neutrophilia independently predicts death in tuberculosis. Eur. Respir. J..

[5] van Kooyk Y, Rabinovich GA (2008). Protein-glycan interactions in the control of innate and adaptive immune responses. Nat. Immunol..

[6] Pinho SS, Reis CA (2015). Glycosylation in cancer: mechanisms and clinical implications. Nat. Rev. Cancer.

[7] Sperandio M, Gleissner CA, Ley K (2009). Glycosylation in immune cell trafficking. Immunol. Rev..

[8] Maas SL, Soehnlein O, Viola JR (2018). Organ-specific mechanisms of transendothelial neutrophil migration in the lung, liver, kidney, and aorta. Front. Immunol..

[9] Rossaint J, Zarbock A (2013). Tissue-specific neutrophil recruitment into the lung, liver, and kidney. J. Innate Immun..

[10] Bartunkova J (2000). Reduced phagocytic activity of polymorphonuclear leukocytes in alpha(1,3) fucosyltransferase VII-deficient mice. APMIS.

[11] Ellies LG (1998). Core 2 oligosaccharide biosynthesis distinguishes between selectin ligands essential for leukocyte homing and inflammation. Immunity.

[12] Duarte, H. O. et al. Mucin-type O-glycosylation in gastric carcinogenesis. *Biomolecules*. 10.3390/biom6030033 (2016). 10.3390/biom6030033 PMC5039419 27409642

[13] Symmes BA, Stefanski AL, Magin CM, Evans CM (2018). Role of mucins in lung homeostasis: regulated expression and biosynthesis in health and disease. Biochem. Soc. Trans..

[14] Tan FY, Tang CM, Exley RM (2015). Sugar coating: bacterial protein glycosylation and host-microbe interactions. Trends Biochem. Sci..

[15] Linden S (2008). Role of ABO secretor status in mucosal innate immunity and H. pylori infection. PLoS Pathog..

[16] Magalhaes A (2015). Helicobacter pylori chronic infection and mucosal inflammation switches the human gastric glycosylation pathways. Biochim. Biophys. Acta.

[17] Marcos NT (2008). Helicobacter pylori induces beta3GnT5 in human gastric cell lines, modulating expression of the SabA ligand sialyl-Lewis x. J. Clin. Invest..

[18] Navabi N, Johansson ME, Raghavan S, Linden SK (2013). Helicobacter pylori infection impairs the mucin production rate and turnover in the murine gastric mucosa. Infect. Immun..

[19] Schreiber T (2006). Selectin ligand-independent priming and maintenance of T cell immunity during airborne tuberculosis. J. Immunol..

[20] Ehlers S, Schreiber T, Dunzendorfer A, Lowe JB, Holscher C (2009). Fucosyltransferase IV and VII-directed selectin ligand function determines long-term survival in experimental tuberculosis. Immunobiology.

[21] Singhania A (2019). Transcriptional profiling unveils type I and II interferon networks in blood and tissues across diseases. Nat. Commun..

[22] Nandi B, Behar SM (2011). Regulation of neutrophils by interferon-gamma limits lung inflammation during tuberculosis infection. J. Exp. Med..

[23] Eash KJ, Means JM, White DW, Link DC (2009). CXCR4 is a key regulator of neutrophil release from the bone marrow under basal and stress granulopoiesis conditions. Blood.

[24] Martin C (2003). Chemokines acting via CXCR2 and CXCR4 control the release of neutrophils from the bone marrow and their return following senescence. Immunity.

[25] Berry MP (2010). An interferon-inducible neutrophil-driven blood transcriptional signature in human tuberculosis. Nature.

[26] Singhania A (2018). A modular transcriptional signature identifies phenotypic heterogeneity of human tuberculosis infection. Nat. Commun..

[27] Vashakidze S (2017). Retrospective study of clinical and lesion characteristics of patients undergoing surgical treatment for Pulmonary Tuberculosis in Georgia. Int. J. Infect. Dis..

[28] Johnson JL, Jones MB, Ryan SO, Cobb BA (2013). The regulatory power of glycans and their binding partners in immunity. Trends Immunol..

[29] Weninger W (2000). Specialized contributions by alpha(1,3)-fucosyltransferase-IV and FucT-VII during leukocyte rolling in dermal microvessels. Immunity.

[30] Maly P (1996). The alpha(1,3)fucosyltransferase Fuc-TVII controls leukocyte trafficking through an essential role in L-, E-, and P-selectin ligand biosynthesis. Cell.

[31] Kolaczkowska E, Kubes P (2013). Neutrophil recruitment and function in health and inflammation. Nat. Rev. Immunol..

[32] Mizgerd JP (1996). Selectins and neutrophil traffic: margination and Streptococcus pneumoniae-induced emigration in murine lungs. J. Exp. Med..

[33] Broide DH (2002). Core 2 oligosaccharides mediate eosinophil and neutrophil peritoneal but not lung recruitment. Am. J. Physiol. Lung Cell Mol. Physiol..

[34] Nouailles G (2014). CXCL5-secreting pulmonary epithelial cells drive destructive neutrophilic inflammation in tuberculosis. J. Clin. Invest..

[35] Isa F (2018). Mass spectrometric identification of urinary biomarkers of pulmonary tuberculosis. EBioMedicine.

[36] Blischak JD, Tailleux L, Mitrano A, Barreiro LB, Gilad Y (2015). Mycobacterial infection induces a specific human innate immune response. Sci. Rep..

[37] Mahdavi J (2002). Helicobacter pylori SabA adhesin in persistent infection and chronic inflammation. Science.

[38] Cagnoni AJ, Perez Saez JM, Rabinovich GA, Marino KV (2016). Turning-off signaling by siglecs, selectins, and galectins: chemical inhibition of glycan-dependent interactions in cancer. Front. Oncol..

[39] Bhatt, K. et al. A nonribosomal peptide synthase gene driving virulence in *Mycobacterium tuberculosis*. *mSphere*. 10.1128/mSphere.00352-18 (2018). 10.1128/mSphere.00352-18 PMC6211224 30381350

[40] Moreira-Teixeira L (2016). Type I IFN inhibits alternative macrophage activation during Mycobacterium tuberculosis infection and leads to enhanced protection in the absence of IFN-gamma signaling. J. Immunol..

[41] Schneider CA, Rasband WS, Eliceiri KW (2012). NIH Image to ImageJ: 25 years of image analysis. Nat. Methods.

[42] Schindelin J (2012). Fiji: an open-source platform for biological-image analysis. Nat. Methods.

[43] Beck TC, Gomes AC, Cyster JG, Pereira JP (2014). CXCR4 and a cell-extrinsic mechanism control immature B lymphocyte egress from bone marrow. J. Exp. Med..

[44] Reutershan J (2006). Critical role of endothelial CXCR2 in LPS-induced neutrophil migration into the lung. J. Clin. Invest..

[45] Radu, M. & Chernoff, J. An in vivo assay to test blood vessel permeability. *J. Vis. Exp*. 10.3791/50062 (2013). 10.3791/50062 PMC3639515 23524912

[46] Ewels P, Magnusson M, Lundin S, Kaller M (2016). MultiQC: summarize analysis results for multiple tools and samples in a single report. Bioinformatics.

[47] Bolger AM, Lohse M, Usadel B (2014). Trimmomatic: a flexible trimmer for Illumina sequence data. Bioinformatics.

[48] Kim D, Langmead B, Salzberg SL (2015). HISAT: a fast spliced aligner with low memory requirements. Nat. Methods.

[49] Pertea M (2015). StringTie enables improved reconstruction of a transcriptome from RNA-seq reads. Nat. Biotechnol..

[50] McCarthy DJ, Chen Y, Smyth GK (2012). Differential expression analysis of multifactor RNA-Seq experiments with respect to biological variation. Nucleic Acids Res..

[51] Robinson MD, McCarthy DJ, Smyth G (2010). K. edgeR: a Bioconductor package for differential expression analysis of digital gene expression data. Bioinformatics.

[52] Ritchie ME (2015). limma powers differential expression analyses for RNA-sequencing and microarray studies. Nucleic Acids Res..

[53] Magalhaes A (2009). Fut2-null mice display an altered glycosylation profile and impaired BabA-mediated Helicobacter pylori adhesion to gastric mucosa. Glycobiology.

[54] Kroesen VM (2018). A beneficial effect of low-dose aspirin in a murine model of active tuberculosis. Front. Immunol..

