# Supplementary material for: Deficiency in the glycosyltransferase Gcnt1 increases susceptibility to tuberculosis through a mechanism involving neutrophils
Source: Mucosal Immunol. 2020 Mar 13;13(5):836–48. doi: 10.1038/s41385-020-0277-7 (PMC7434595; doi:10.1038/s41385-020-0277-7)
Supplement: Supplementary file 1 — Supplementary Information [file 41385_2020_277_MOESM1_ESM.pdf]

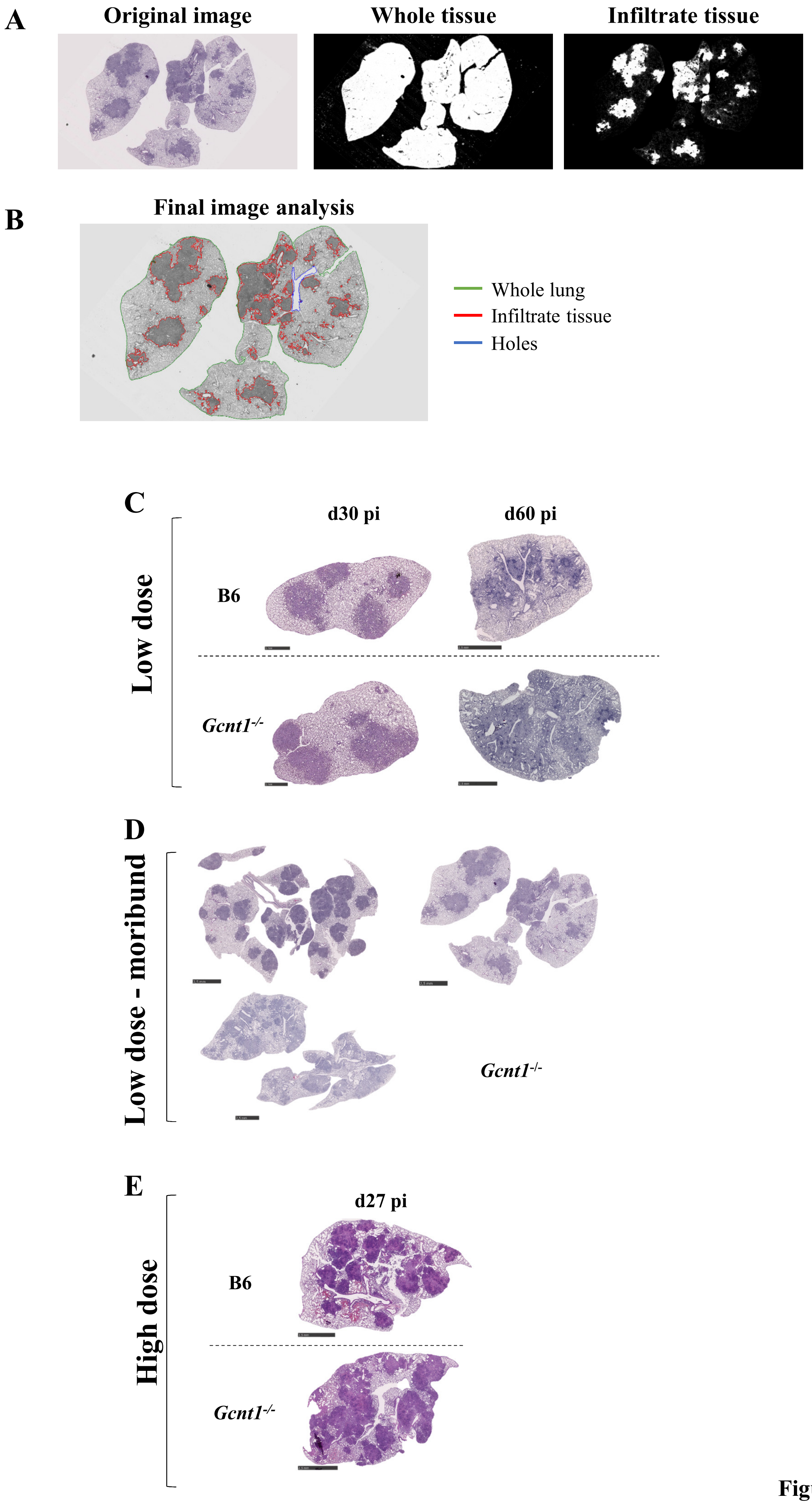
A
Original image
Whole tissue
Infiltrate tissue
B
Final image analysis
Whole lung
Infiltrate tissue
Holes
C
d30 pi
d60 pi
Low dose
B6
Gcnt1-/-
D
Low dose - moribund
Gcnt1-/-
E
d27 pi
High dose
B6
Gcnt1-/-

Figure S1

**Supplemental Figure 1. Lung pathology in C57BL/6 vs *Gcnt1*$^{-/-}$ mice upon aerosol infection with *M. tuberculosis*.** The morphometric analysis was determined using ilastik: Interactive Learning and Segmentation Toolkit (www.ilastik.org/publications.html). (A) Probability maps were obtained for whole lung tissues and for lesion areas and (B) analysed using the CellProfiler Analyst software (version 3.1.5). Whole lung tissue (green line), lung infiltrates (red line) and lung holes (blue line) are identified. Areas were quantified and the percentage of infiltrates per lung calculated. (C,D,E) H&E staining of whole lung sections are shown for mice representative of each experimental group. Scale bar corresponds to 1 mm (for low dose d30pi) and 2.5 mm (for low dose d60pi) (C); 5 mm (low dose moribund) (D); and 2.5 mm (for high dose d27pi) (E).

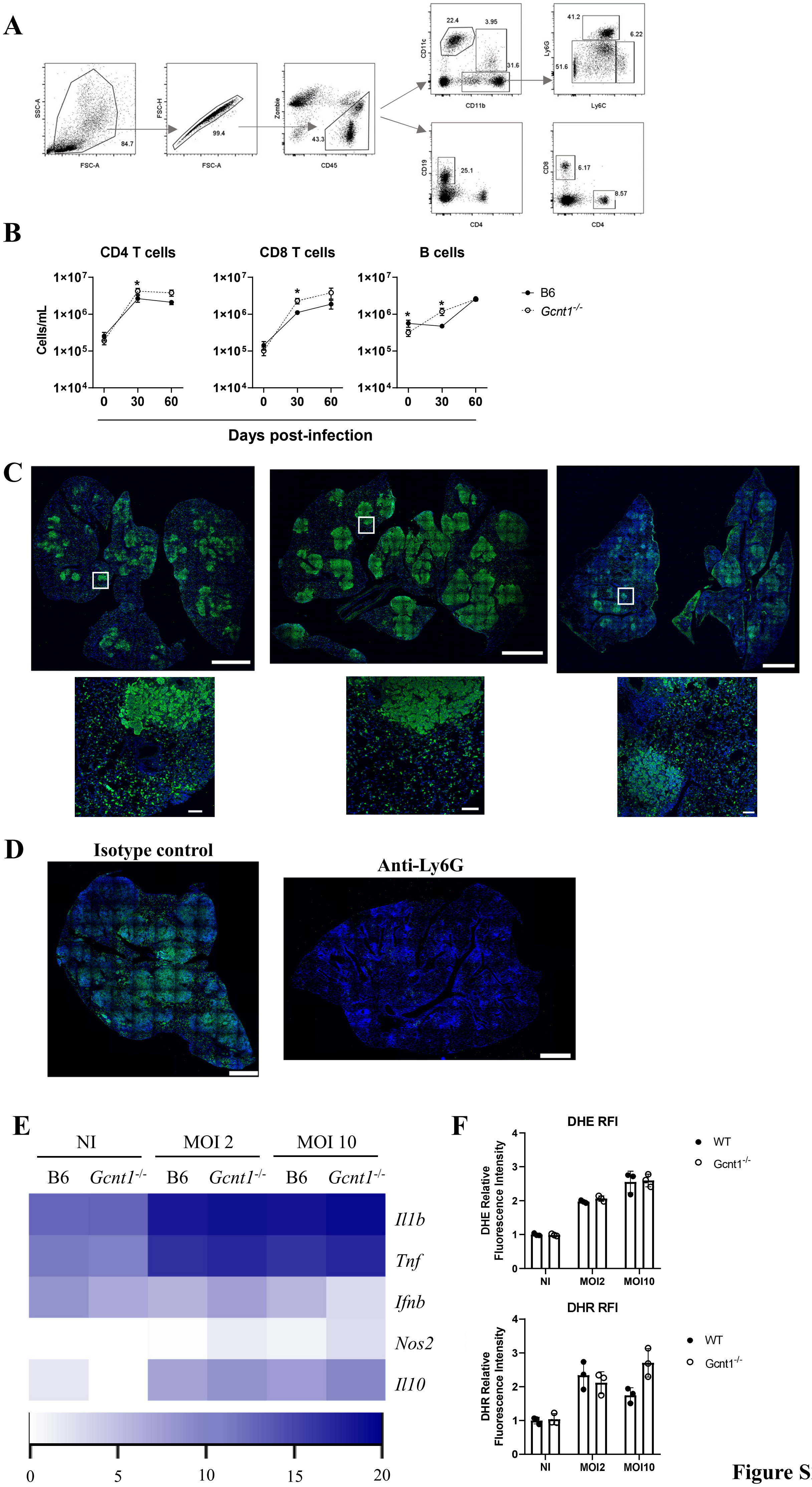
A
SSC-A
FSC-A
84.7
FSC-H
99.4
Zombie
CD45
43.3
CD11c
CD11b
22.4
3.95
31.6
Ly6G
Ly6C
41.2
6.22
51.6
CD19
CD4
25.1
CD8
6.17
8.57
B
CD4 T cells
CD8 T cells
B cells
Cells/mL
Days post-infection
B6
Gcnt1-/-
C
D
Isotype control
Anti-Ly6G
E
NI
MOI 2
MOI 10
B6
Gcnt1-/-
Il1b
Tnf
Ifnb
Nos2
Il10
0
5
10
15
20
F
DHE RFI
DHE Relative Fluorescence Intensity
WT
Gcnt1-/-
NI
MOI2
MOI10
DHR RFI
DHR Relative Fluorescence Intensity

Figure S2

**Supplemental Figure 2.** (A) Gating strategies to detect immune cell populations in the lung of C57BL/6 and *Gcnt1*$^{-/-}$ mice. (B) At the indicated time-points post-infection, the lungs of C57BL/6 (B6; black circles) or *Gcnt1*$^{-/-}$ (open circles) mice infected by aerosol with a low dose of *Mtb* strain HN878 were harvested and a cellular suspension prepared. The indicated immune cell populations were determined by flow cytometry. Each dot represents the Mean±SEM for 10 animals in 2 independent experiments. Non-infected animals (day 0) were used as controls. Statistical analysis was performed with unpaired two-tailed Mann-Whitney test for each time point. * refer to statistic differences between B6 or *Gcnt1*$^{-/-}$ mice. *, $p<0.05$. (C) Immunofluorescence detection of MPO (green) in the lungs of *Gcnt1*$^{-/-}$ moribund mice. Sections were counterstained with DAPI (blue). Scale bars correspond to 2.5 mm on whole lung images and 100 µm on close ups. (D) Representative images of lung sections of infected *Gcnt1*$^{-/-}$ mice that have received isotype control (left) or anti-Ly6G (right) stained with MPO and DAPI. Scale bars correspond to 1 mm. (E,F) Neutrophils were purified from the BM of C57BL/6 (B6) or *Gcnt1*$^{-/-}$ animals and infected in vitro with *Mtb* HN878 at a moi of 2 or 10 as indicated. Four h post-infection, (E) the RNA of infected cells was harvested, converted to cDNA and the expression of the indicated genes measured by real-time PCR. Represented is a heatmap of log2 relative expression of depicted genes. (F) Detection of reactive oxygen species (ROS) production by neutrophil using dihydroethidium (DHE) and Dihydrorhodamine 123 (DHR) probes. Represented are the Mean±SEM. Each dot represents one well of the experiment.

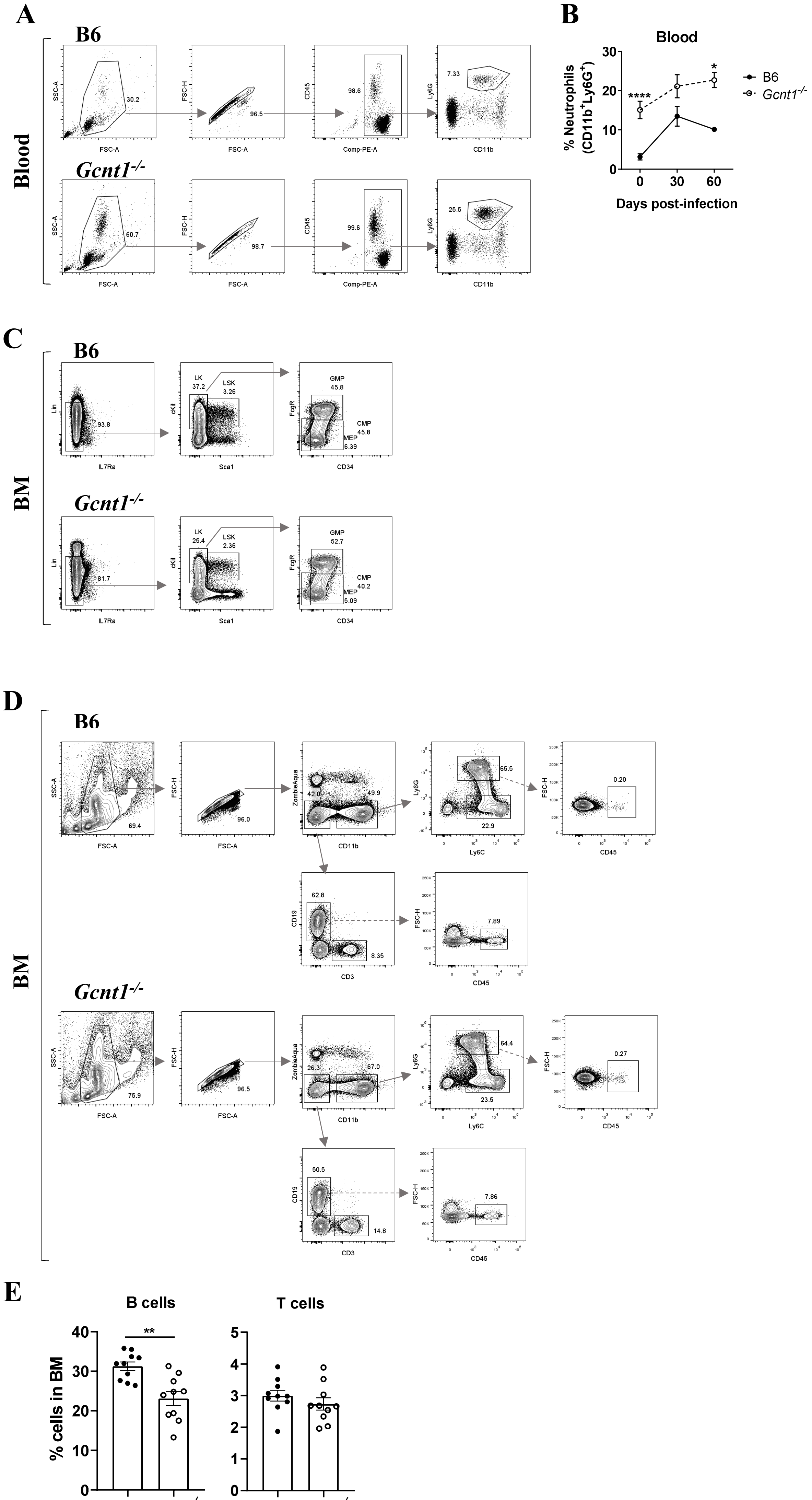
A
B6
Blood
Gcnt1-/-
SSC-A
FSC-A
FSC-H
CD45
Comp-PE-A
Ly6G
CD11b
30.2
96.5
98.6
7.33
60.7
98.7
99.6
25.5
B
Blood
% Neutrophils (CD11b+Ly6G+)
Days post-infection
B6
Gcnt1-/-
****
*
C
B6
BM
Gcnt1-/-
Lin
IL7Ra
cKit
Sca1
FcgR
CD34
LK
LSK
GMP
CMP
MEP
93.8
37.2
3.26
45.8
45.8
6.39
81.7
25.4
2.36
52.7
40.2
5.09
D
B6
BM
Gcnt1-/-
ZombieAqua
CD11b
Ly6C
CD19
CD3
69.4
96.0
42.0
49.9
65.5
22.9
0.20
62.8
8.35
7.89
75.9
96.5
26.3
67.0
64.4
23.5
0.27
50.5
14.8
7.86
E
B cells
T cells
% cells in BM
**
B6
Gcnt1-/-

Figure S3

**Supplemental Figure 3.** (A,C,D) Gating strategies to detect immune cell populations in the blood and BM of C57BL/6 and *Gcnt1*$^{-/-}$ mice. (B) At the indicated time-points post-infection, the blood of C57BL/6 (B6; black circles) or *Gcnt1*$^{-/-}$ (open circles) mice infected by aerosol with a low dose of Mtb strain HN878 was harvested and the frequency of neutrophils determined by flow cytometry. Non-infected mice were used as controls on day 0. (E) Frequencies of B and T cells present in the BM of uninfected C57BL/6 (B6; black circles) or *Gcnt1*$^{-/-}$ (open circles) mice were determined by flow cytometry. (B,E) Represented are the Mean±SEM. Each dot represents a mouse. Statistical analysis was performed with unpaired two-tailed Mann-Whitney test for each time point (B) or Student's t-test (E). * refer to statistic differences between B6 or *Gcnt1*$^{-/-}$ mice. *, $p<0.05$; **, $p<0.01$; ****, $p<0.0001$.

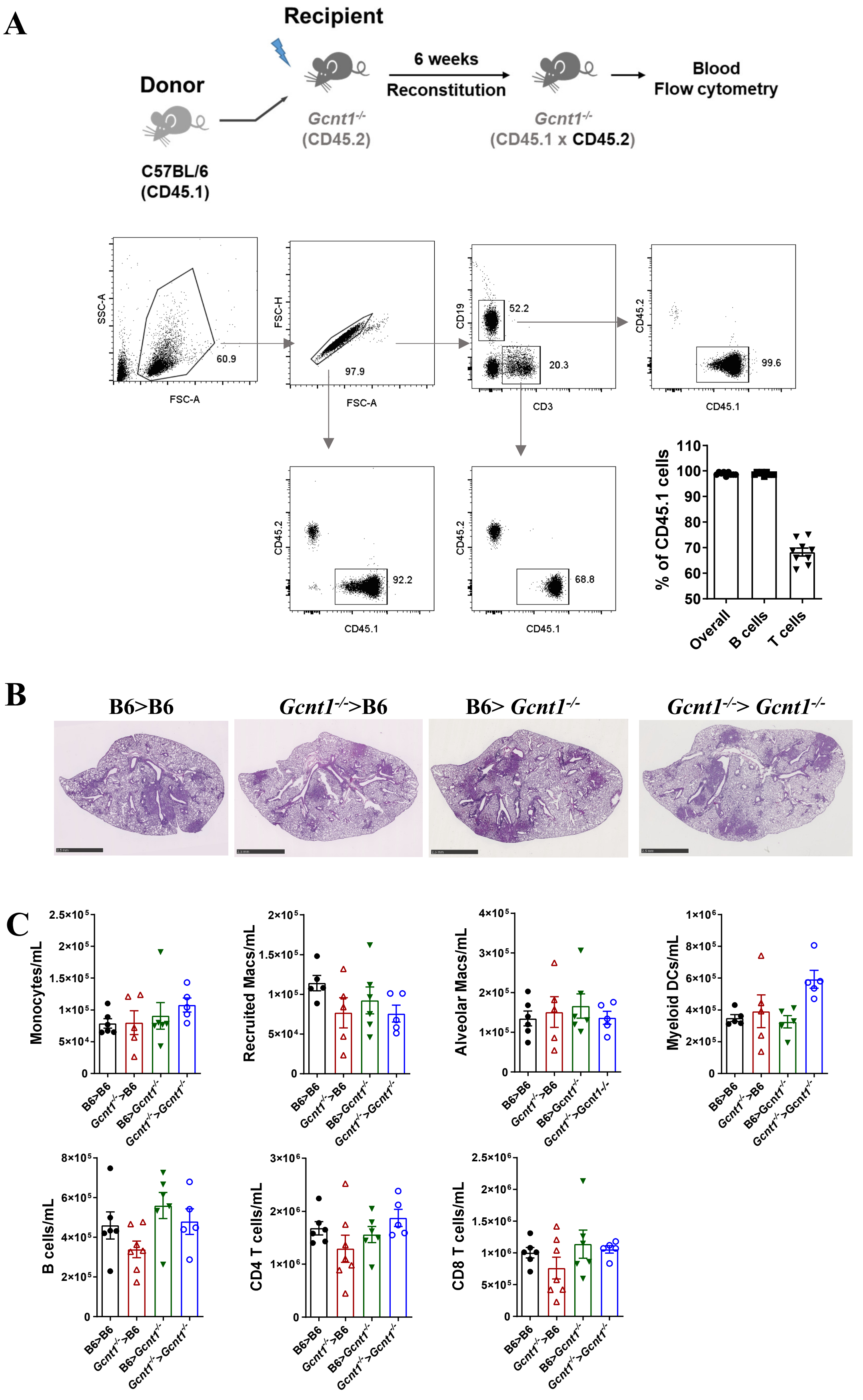


**Supplemental Figure 4. Global characterization of the cellular content of BM reconstituted mice, pre- and post-infection with *M. tuberculosis*.** (A) A control group was included to assess the efficiency of the BM reconstitution model through flow cytometry in whole blood analysis. (B) H&E staining of whole lung sections are shown for one mouse representative of each experimental group. Scale bar corresponds to 2.5 mm. (C) The indicated immune cell populations were determined by flow cytometry for each experimental group. (A, C) Represented are mean±SEM for each group; individual dots refer to individual mice. Statistical analysis was performed as in Figure 2.

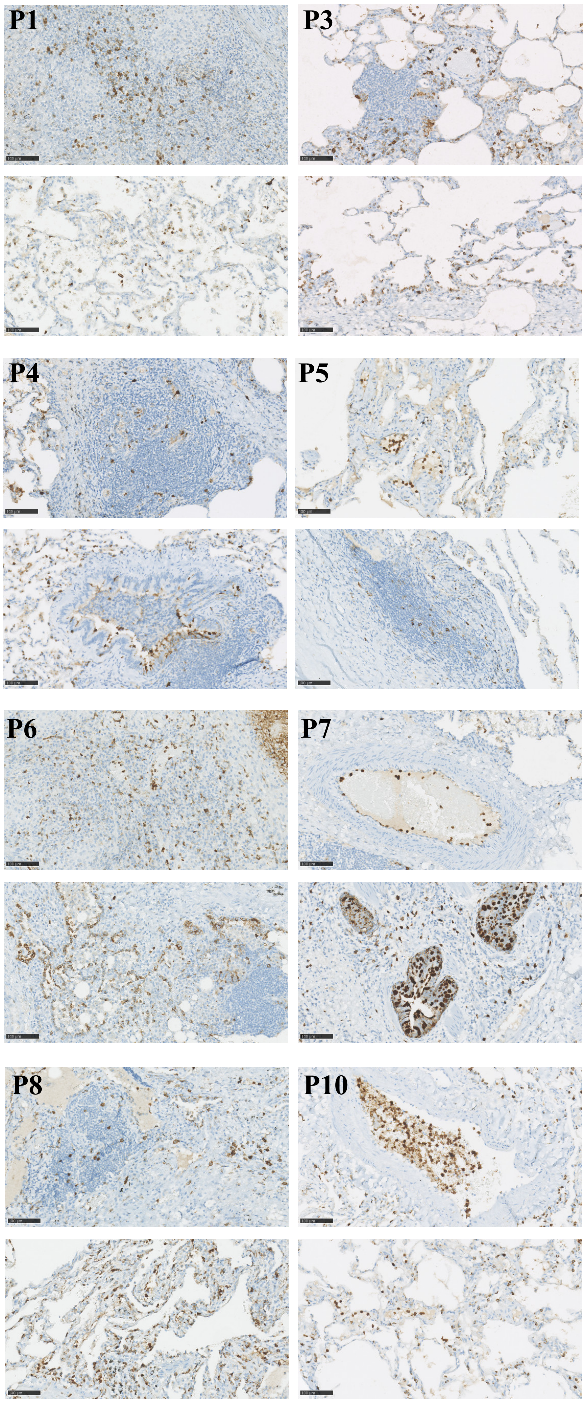


**Supplemental Figure 5. sLe$^x$ detection in lung sections of TB patients.** Lung sections of TB patients who underwent therapeutic surgery were stained for sLe$^x$ as in Figure 5. Scale bar corresponds to 100 µm.

**Table S1.** Log fold change and significance values for differential expression of glycosyltransferase coding genes in Berry London, Berry Leicester and Berry South Africa datasets (Singhania et al., 2018) #.

| GENE NAME | BERRY LONDON | | | | | | | | BERRY LEICESTER (progressors) | | | | | | | | BERRY SOUTH AFRICA | |
|---|---|---|---|---|---|---|---|---|---|---|---|---|---|---|---|---|---|---|
| | ACTIVE vs CONTROL | | ACTIVE vs LATENT | | LATENT vs CONTROL | | Multiple Testing | | ACTIVE vs CONTROL | | ACTIVE vs LATENT | | LATENT vs CONTROL | | Multiple Testing | | ACTIVE vs LATENT | |
| | *Log(FC*)* | *P-value* | *Log(FC)* | *P-value* | *Log(FC)* | *P-value* | *P-value* | *FDR** | *Log(FC)* | *P-value* | *Log(FC)* | *P-value* | *Log(FC)* | *P-value* | *P-value* | *FDR* | *Log(FC)* | *P-value* |
| *A4GALT* | 0,57039 | 0,31023 | 0,35805 | 0,45416 | 0,21235 | 0,70448 | 0,56005 | 0,68750 | 0,62562 | 0,02803 | 0,57112 | 0,04575 | 0,05450 | 0,84997 | 0,05076 | 0,08362 | 0,70089 | 0,21311 |
| *A4GNT* | | | | | | | | | | | | | | | | | | |
| *ABO* | -0,21185 | 0,61946 | -0,34962 | 0,33799 | 0,13778 | 0,74655 | 0,62642 | 0,74071 | -0,02411 | 0,91770 | -0,23454 | 0,31821 | 0,21042 | 0,37713 | 0,55251 | 0,61835 | 0,30780 | 0,62837 |
| *ALG1* | -0,05174 | 0,73763 | -0,20705 | 0,11980 | 0,15531 | 0,31655 | 0,27604 | 0,43549 | -0,22784 | 0,00122 | -0,25548 | 0,00033 | 0,02764 | 0,69547 | 0,00039 | 0,00113 | -0,42005 | 0,00305 |
| *ALG10* | -0,21099 | 0,52512 | -0,28090 | 0,32243 | 0,06991 | 0,83296 | 0,59371 | 0,71440 | -0,38126 | 0,07942 | -0,49217 | 0,02475 | 0,11091 | 0,61513 | 0,06007 | 0,09670 | -0,10956 | 0,80242 |
| *ALG11* | 0,34716 | 0,09996 | 0,12256 | 0,49149 | 0,22460 | 0,28380 | 0,25527 | 0,41415 | -0,27900 | 0,03756 | -0,16007 | 0,23303 | -0,11893 | 0,38185 | 0,11181 | 0,16413 | 0,16132 | 0,32799 |
| *ALG12* | 0,07734 | 0,45597 | -0,14245 | 0,11057 | 0,21980 | 0,03730 | 0,08438 | 0,20195 | -0,08510 | 0,15741 | -0,10045 | 0,09728 | 0,01535 | 0,80183 | 0,19766 | 0,26531 | -0,22336 | 0,10294 |
| *ALG13* | -0,24349 | 0,03734 | -0,17424 | 0,07890 | -0,06925 | 0,54654 | 0,07270 | 0,18337 | -0,55063 | 0,00000 | -0,35439 | 0,00104 | -0,19624 | 0,07000 | 0,00000 | 0,00001 | -0,19180 | 0,06613 |
| *ALG14* | 0,29644 | 0,19204 | -0,04360 | 0,82066 | 0,34004 | 0,13547 | 0,29324 | 0,45345 | -0,25102 | 0,16972 | -0,32257 | 0,07979 | 0,07156 | 0,70025 | 0,17951 | 0,24473 | -0,46084 | 0,13651 |
| *ALG2* | 0,36290 | 0,11678 | -0,17607 | 0,36863 | 0,53898 | 0,02148 | 0,06903 | 0,17771 | -0,45612 | 0,00879 | -0,29345 | 0,09141 | -0,16267 | 0,35469 | 0,02867 | 0,05102 | -0,02672 | 0,84910 |
| *ALG3* | -0,10849 | 0,22443 | -0,13669 | 0,07491 | 0,02820 | 0,75068 | 0,17921 | 0,32851 | 0,15594 | 0,01707 | 0,01403 | 0,82944 | 0,14191 | 0,03293 | 0,03280 | 0,05731 | -0,29395 | 0,01365 |
| *ALG5**** | | | | | | | | | | | | | | | | | | |
| *ALG6* | 0,01220 | 0,94209 | -0,01123 | 0,93751 | 0,02343 | 0,88905 | 0,99001 | 0,99293 | -0,13884 | 0,29120 | -0,18949 | 0,15248 | 0,05065 | 0,70528 | 0,32873 | 0,40474 | 0,38508 | 0,02606 |
| *ALG8* | -0,18025 | 0,09394 | 0,02318 | 0,79815 | -0,20343 | 0,05957 | 0,13922 | 0,27977 | -0,04366 | 0,47449 | -0,14382 | 0,02008 | 0,10016 | 0,10881 | 0,05957 | 0,09598 | -0,20951 | 0,05970 |
| *ALG9* | -0,38475 | 0,04557 | -0,62166 | 0,00028 | 0,23691 | 0,21317 | 0,00120 | 0,01263 | -0,94154 | 0,00021 | -0,51653 | 0,03999 | -0,42501 | 0,09491 | 0,00099 | 0,00260 | -1,04708 | 0,00303 |
| *B3GALNT1* | 0,73749 | 0,10943 | 1,14617 | 0,00445 | -0,40868 | 0,37123 | 0,01577 | 0,06841 | -0,08526 | 0,72442 | -0,17320 | 0,47645 | 0,08794 | 0,72136 | 0,77541 | 0,81628 | 0,76473 | 0,13222 |
| *B3GALNT2* | -0,12022 | 0,65585 | 0,03000 | 0,89615 | -0,15022 | 0,57781 | 0,84931 | 0,90139 | -0,87162 | 0,00218 | -0,33448 | 0,23585 | -0,53715 | 0,06143 | 0,00846 | 0,01750 | -0,59618 | 0,15558 |
| *B3GALT1* | | | | | | | | | | | | | | | | | | |
| *B3GALT2* | -0,50849 | 0,24659 | -0,81426 | 0,03206 | 0,30577 | 0,48427 | 0,09548 | 0,21873 | -0,91424 | 0,00092 | -0,86622 | 0,00176 | -0,04802 | 0,86204 | 0,00095 | 0,00252 | -1,06423 | 0,01271 |
| *B3GALT4* | 0,38556 | 0,00110 | 0,08940 | 0,35329 | 0,29616 | 0,01061 | 0,00403 | 0,02824 | 0,50939 | 0,00000 | 0,43863 | 0,00000 | 0,07076 | 0,33644 | 0,00000 | 0,00000 | -0,00769 | 0,93835 |
| *B3GALT5* | | | | | | | | | | | | | | | | | | |
| *B3GALT6* | -0,02312 | 0,83855 | -0,09812 | 0,31283 | 0,07500 | 0,50950 | 0,57953 | 0,70302 | -0,05854 | 0,35845 | -0,15684 | 0,01521 | 0,09830 | 0,13135 | 0,04982 | 0,08225 | -0,37194 | 0,00567 |
| *B3GAT1* | -0,21542 | 0,58621 | -0,26392 | 0,43488 | 0,04849 | 0,90235 | 0,71548 | 0,80837 | -0,78690 | 0,00000 | -0,62673 | 0,00019 | -0,16016 | 0,33680 | 0,00001 | 0,00003 | -0,58480 | 0,02072 |
| *B3GAT2* | 0,22501 | 0,34125 | 0,52972 | 0,01046 | -0,30471 | 0,19898 | 0,03607 | 0,11751 | -0,11376 | 0,62200 | -0,07831 | 0,73561 | -0,03545 | 0,88017 | 0,87950 | 0,90355 | -0,22721 | 0,47174 |
| *B3GAT3* | 0,00470 | 0,96730 | -0,20800 | 0,03705 | 0,21271 | 0,06772 | 0,06714 | 0,17464 | 0,06741 | 0,34923 | -0,00301 | 0,96682 | 0,07041 | 0,33759 | 0,54975 | 0,61595 | -0,35932 | 0,00122 |

| | | | | | | | | | | | | | | | | | | |
|---|---|---|---|---|---|---|---|---|---|---|---|---|---|---|---|---|---|---|
| *B3GLCT* | -0,12021 | 0,56744 | -0,43435 | 0,01801 | 0,31414 | 0,13841 | 0,05281 | 0,14937 | -0,53909 | 0,00009 | -0,55446 | 0,00006 | 0,01538 | 0,91062 | 0,00003 | 0,00011 | -0,25311 | 0,43459 |
| *B3GNT2* | 0,22354 | 0,07974 | 0,24191 | 0,02743 | -0,01837 | 0,88390 | 0,05922 | 0,16093 | -0,44304 | 0,00268 | -0,22778 | 0,12061 | -0,21526 | 0,14791 | 0,01083 | 0,02179 | 0,51060 | 0,00007 |
| *B3GNT3* | -0,21972 | 0,65547 | -0,58414 | 0,16753 | 0,36441 | 0,46001 | 0,37761 | 0,53435 | -0,54224 | 0,03937 | -0,20038 | 0,44607 | -0,34186 | 0,20076 | 0,11466 | 0,16764 | -0,82417 | 0,10067 |
| *B3GNT4* | 0,40945 | 0,49433 | 0,64640 | 0,20811 | -0,23695 | 0,69206 | 0,44404 | 0,59186 | 0,10991 | 0,70466 | 0,28293 | 0,33236 | -0,17302 | 0,55854 | 0,62089 | 0,68092 | 0,62287 | 0,37579 |
| *B3GNT5* | 0,36312 | 0,28648 | 0,09739 | 0,73628 | 0,26572 | 0,43431 | 0,55887 | 0,68651 | 0,11604 | 0,62917 | 0,45142 | 0,06304 | -0,33538 | 0,17221 | 0,15787 | 0,21960 | 1,28409 | 0,00001 |
| *B3GNT6* | | | | | | | | | | | | | | | | | | |
| *B3GNT7* | -0,48748 | 0,08099 | -0,75712 | 0,00204 | 0,26964 | 0,32985 | 0,00759 | 0,04252 | -1,31728 | 0,00000 | -0,97472 | 0,00002 | -0,34256 | 0,12451 | 0,00000 | 0,00000 | -1,65116 | 0,00001 |
| *B3GNT8* | 0,38123 | 0,04214 | 0,44410 | 0,00627 | -0,06288 | 0,73286 | 0,01547 | 0,06750 | 0,69268 | 0,00000 | 0,64493 | 0,00000 | 0,04775 | 0,68580 | 0,00000 | 0,00000 | 0,39677 | 0,02611 |
| *B3GNT9* | 0,07367 | 0,62076 | -0,07528 | 0,55343 | 0,14895 | 0,31876 | 0,59554 | 0,71579 | -0,12615 | 0,36313 | 0,03027 | 0,82791 | -0,15642 | 0,26905 | 0,49890 | 0,56929 | 0,15581 | 0,27087 |
| *B3GNTL1* | 0,13932 | 0,29481 | 0,11710 | 0,30176 | 0,02222 | 0,86665 | 0,46653 | 0,61051 | 0,22988 | 0,00935 | 0,36406 | 0,00006 | -0,13419 | 0,13390 | 0,00023 | 0,00070 | 0,13214 | 0,37943 |
| *B4GALNT1* | | | | | | | | | | | | | | | | | | |
| *B4GALNT2* | | | | | | | | | | | | | | | | | | |
| *B4GALNT3* | 0,28908 | 0,51813 | -0,06939 | 0,85543 | 0,35847 | 0,42341 | 0,71254 | 0,80616 | 0,23212 | 0,41350 | 0,35077 | 0,21960 | -0,11865 | 0,68161 | 0,45450 | 0,52710 | -0,16164 | 0,77411 |
| *B4GALNT4* | -0,04861 | 0,94813 | -0,45794 | 0,47342 | 0,40932 | 0,58436 | 0,74463 | 0,82958 | -0,36165 | 0,26396 | -0,64750 | 0,04760 | 0,28586 | 0,38612 | 0,13751 | 0,19558 | -0,20935 | 0,73331 |
| ***B4GALT1**** | 0,06210 | 0,41014 | 0,06818 | 0,28993 | -0,00608 | 0,93552 | 0,52268 | 0,65724 | -0,03414 | 0,45815 | 0,02887 | 0,53240 | -0,06301 | 0,18016 | 0,40503 | 0,48023 | 0,17401 | 0,03542 |
| ***B4GALT2*** | -0,41195 | 0,20503 | -0,56334 | 0,04445 | 0,15139 | 0,63929 | 0,11871 | 0,25256 | -0,04197 | 0,70863 | -0,16457 | 0,14622 | 0,12260 | 0,28513 | 0,32323 | 0,39962 | -0,67867 | 0,04087 |
| ***B4GALT3*** | 0,06256 | 0,66270 | -0,09274 | 0,44905 | 0,15530 | 0,28098 | 0,52753 | 0,66138 | 0,10865 | 0,17534 | -0,02435 | 0,76186 | 0,13300 | 0,10418 | 0,22000 | 0,29016 | -0,35101 | 0,02178 |
| ***B4GALT4*** | 0,18523 | 0,20546 | 0,16851 | 0,17723 | 0,01672 | 0,90835 | 0,29956 | 0,45971 | 0,00754 | 0,92792 | -0,00668 | 0,93649 | 0,01422 | 0,86715 | 0,98604 | 0,98897 | 0,34156 | 0,06936 |
| *B4GALT5* | 0,61451 | 0,00059 | 0,63465 | 0,00005 | -0,02014 | 0,90527 | 0,00006 | 0,00183 | 0,78404 | 0,00000 | 0,81922 | 0,00000 | -0,03518 | 0,74924 | 0,00000 | 0,00000 | 1,18450 | 0,00000 |
| ***B4GALT6*** | 0,34415 | 0,35295 | -0,46655 | 0,14212 | 0,81070 | 0,03148 | 0,08190 | 0,19789 | -1,05285 | 0,00003 | -0,62501 | 0,01197 | -0,42784 | 0,08811 | 0,00014 | 0,00045 | -1,13990 | 0,00254 |
| *B4GALT7* | -0,29553 | 0,03755 | -0,26684 | 0,02803 | -0,02869 | 0,83690 | 0,04120 | 0,12763 | 0,01662 | 0,87361 | 0,04874 | 0,64268 | -0,03212 | 0,76303 | 0,89517 | 0,91653 | -0,48443 | 0,00022 |
| *B4GAT1* | -0,72169 | 0,00002 | -0,61347 | 0,00002 | -0,10822 | 0,48759 | 0,00001 | 0,00041 | -0,77503 | 0,00000 | -0,77189 | 0,00000 | -0,00314 | 0,97706 | 0,00000 | 0,00000 | -0,79299 | 0,01509 |
| *C1GALT1* | 0,27302 | 0,31977 | 0,36283 | 0,12339 | -0,08981 | 0,74248 | 0,28256 | 0,44232 | 0,36985 | 0,00075 | 0,23343 | 0,03240 | 0,13642 | 0,21543 | 0,00289 | 0,00677 | 0,80138 | 0,00299 |
| *C1GALT1C1* | 1,32903 | 0,00103 | 0,17262 | 0,59994 | 1,15641 | 0,00388 | 0,00279 | 0,02220 | -0,06483 | 0,79419 | 0,05412 | 0,82849 | -0,11894 | 0,63885 | 0,89506 | 0,91650 | 0,12183 | 0,81429 |
| *CERCAM* | 0,22858 | 0,61694 | 0,24217 | 0,53457 | -0,01360 | 0,97623 | 0,79571 | 0,86433 | -0,32466 | 0,05579 | 0,01919 | 0,90992 | -0,34385 | 0,04705 | 0,07998 | 0,12351 | -0,68062 | 0,07109 |
| *CHPF* | -0,10004 | 0,62237 | -0,15852 | 0,36143 | 0,05848 | 0,77326 | 0,65274 | 0,76050 | -0,08362 | 0,43354 | -0,22333 | 0,03860 | 0,13972 | 0,20009 | 0,11332 | 0,16595 | -0,81211 | 0,03380 |
| *CHPF2* | 0,40953 | 0,00211 | 0,18744 | 0,08902 | 0,22210 | 0,08580 | 0,00796 | 0,04401 | 0,29864 | 0,00000 | 0,34571 | 0,00000 | -0,04707 | 0,41894 | 0,00000 | 0,00000 | -0,02856 | 0,77065 |
| *CHSY1* | 0,44067 | 0,00429 | 0,39777 | 0,00261 | 0,04290 | 0,77301 | 0,00268 | 0,02166 | 0,12468 | 0,34672 | 0,15618 | 0,24135 | -0,03150 | 0,81541 | 0,45848 | 0,53085 | 0,95839 | 0,00000 |
| *CHSY3* | | | | | | | | | | | | | | | | | 0,08346 | 0,86422 |
| *COLGALT1* | 0,16190 | 0,06080 | 0,04214 | 0,56156 | 0,11976 | 0,16245 | 0,16459 | 0,31103 | 0,31693 | 0,00002 | 0,35602 | 0,00000 | -0,03909 | 0,59470 | 0,00000 | 0,00001 | -0,12262 | 0,21992 |
| *COLGALT2* | -0,70591 | 0,05962 | -0,97414 | 0,00294 | 0,26824 | 0,46806 | 0,00959 | 0,04954 | -1,04476 | 0,00000 | -1,01918 | 0,00000 | -0,02558 | 0,88989 | 0,00000 | 0,00000 | -0,85871 | 0,09643 |

| | | | | | | | | | | | | | | | | | | |
|---|---|---|---|---|---|---|---|---|---|---|---|---|---|---|---|---|---|---|
| *CSGALNACT1* | 0,14454 | 0,51453 | 0,11027 | 0,55968 | 0,03427 | 0,87696 | 0,76225 | 0,84163 | -0,67295 | 0,00005 | -0,33593 | 0,03987 | -0,33702 | 0,04206 | 0,00026 | 0,00079 | 0,11990 | 0,59643 |
| *CSGALNACT2* | 0,32479 | 0,00233 | 0,30180 | 0,00099 | 0,02299 | 0,82217 | 0,00101 | 0,01130 | -0,20965 | 0,09031 | -0,04557 | 0,71296 | -0,16408 | 0,19271 | 0,20779 | 0,27674 | 0,55166 | 0,00002 |
| *DPM1* | -0,11135 | 0,34697 | 0,22020 | 0,03201 | -0,33155 | 0,00656 | 0,01424 | 0,06402 | -0,16761 | 0,12795 | -0,16727 | 0,13071 | -0,00034 | 0,99759 | 0,21173 | 0,28130 | 0,39330 | 0,00172 |
| *DPY19L1* | 0,18683 | 0,24857 | -0,03729 | 0,78594 | 0,22412 | 0,16743 | 0,35944 | 0,51776 | -0,16333 | 0,18193 | -0,26872 | 0,02969 | 0,10539 | 0,39740 | 0,08880 | 0,13512 | 0,29422 | 0,02946 |
| *DPY19L2* | -0,83979 | 0,12713 | -0,74340 | 0,11360 | -0,09639 | 0,85956 | 0,18365 | 0,33398 | | | | | | | | | 0,46317 | 0,43040 |
| *DPY19L3* | 0,68890 | 0,06165 | 0,67195 | 0,03339 | 0,01696 | 0,96273 | 0,05988 | 0,16205 | -0,20959 | 0,45038 | -0,12465 | 0,65499 | -0,08494 | 0,76401 | 0,74813 | 0,79252 | 0,23537 | 0,32267 |
| *EOGT* | 0,03207 | 0,83875 | -0,05215 | 0,69814 | 0,08422 | 0,59348 | 0,85258 | 0,90355 | -0,26599 | 0,11163 | -0,40603 | 0,01626 | 0,14004 | 0,40995 | 0,04924 | 0,08143 | -0,17632 | 0,50512 |
| *EXT1* | 0,30701 | 0,04145 | 0,28986 | 0,02457 | 0,01715 | 0,90761 | 0,04001 | 0,12528 | 0,41104 | 0,00000 | 0,37058 | 0,00001 | 0,04046 | 0,61846 | 0,00000 | 0,00000 | 0,49369 | 0,00135 |
| *EXT2* | 0,10055 | 0,35009 | -0,01554 | 0,86502 | 0,11609 | 0,28129 | 0,52886 | 0,66244 | -0,04560 | 0,46329 | -0,00830 | 0,89427 | -0,03730 | 0,55614 | 0,73945 | 0,78538 | -0,42672 | 0,00366 |
| *EXTL1* | | | | | | | | | | | | | | | | | | |
| *EXTL2* | 0,71369 | 0,03147 | -0,26622 | 0,33861 | 0,97991 | 0,00371 | 0,01350 | 0,06178 | -0,91279 | 0,00025 | -0,51954 | 0,03521 | -0,39325 | 0,11488 | 0,00112 | 0,00290 | -0,68461 | 0,01520 |
| *EXTL3* | 0,05319 | 0,73990 | 0,01322 | 0,92291 | 0,03998 | 0,80293 | 0,94474 | 0,96385 | -0,00761 | 0,95766 | 0,05730 | 0,69078 | -0,06490 | 0,65689 | 0,88810 | 0,91092 | 0,00135 | 0,99331 |
| *FUT1* | | | | | | | | | | | | | | | | | | |
| ***FUT10*** | -0,25617 | 0,35278 | -0,65667 | 0,00670 | 0,40050 | 0,14854 | 0,02361 | 0,08901 | -0,79507 | 0,00009 | -0,71582 | 0,00042 | -0,07925 | 0,69444 | 0,00009 | 0,00031 | -0,78377 | 0,00018 |
| ***FUT11*** | -0,07500 | 0,52379 | -0,18495 | 0,06889 | 0,10994 | 0,35106 | 0,18593 | 0,33640 | -0,37881 | 0,00000 | -0,38541 | 0,00000 | 0,00660 | 0,92058 | 0,00000 | 0,00000 | -0,22837 | 0,05668 |
| *FUT2* | 0,12604 | 0,68184 | -0,48096 | 0,07049 | 0,60700 | 0,05210 | 0,08495 | 0,20277 | -1,09384 | 0,00000 | -1,01839 | 0,00000 | -0,07544 | 0,71930 | 0,00000 | 0,00000 | -1,98438 | 0,00001 |
| ***FUT3*** | | | | | | | | | | | | | | | | | | |
| ***FUT4*** | 0,49005 | 0,00289 | 0,20953 | 0,12379 | 0,28053 | 0,07982 | 0,01104 | 0,05430 | 0,38170 | 0,00000 | 0,40148 | 0,00000 | -0,01978 | 0,70785 | 0,00000 | 0,00000 | 0,33470 | 0,00510 |
| ***FUT5*** | | | | | | | | | | | | | | | | | | |
| ***FUT6*** | | | | | | | | | | | | | | | | | | |
| ***FUT7*** | 0,68346 | 0,00025 | 0,55447 | 0,00045 | 0,12898 | 0,46267 | 0,00016 | 0,00341 | 0,70047 | 0,00000 | 0,62631 | 0,00000 | 0,07416 | 0,50819 | 0,00000 | 0,00000 | 0,41142 | 0,00905 |
| *FUT8* | -0,38464 | 0,00886 | -0,16761 | 0,17125 | -0,21703 | 0,13155 | 0,03069 | 0,10575 | -0,39792 | 0,00032 | -0,43720 | 0,00009 | 0,03928 | 0,72227 | 0,00008 | 0,00027 | -0,36114 | 0,00262 |
| *FUT9* | | | | | | | | | | | | | | | | | | |
| ***GALNT1*** | -0,01743 | 0,88824 | 0,12394 | 0,24412 | -0,14137 | 0,25707 | 0,39457 | 0,54882 | -0,23669 | 0,06089 | -0,12389 | 0,32705 | -0,11279 | 0,37889 | 0,17110 | 0,23505 | 0,14595 | 0,24410 |
| ***GALNT10*** | 0,21599 | 0,18009 | -0,16012 | 0,24298 | 0,37611 | 0,02160 | 0,06863 | 0,17701 | -0,10803 | 0,36939 | -0,02193 | 0,85595 | -0,08609 | 0,48271 | 0,64041 | 0,69851 | -0,21172 | 0,40675 |
| ***GALNT11*** | 0,00776 | 0,92021 | -0,16467 | 0,01521 | 0,17243 | 0,02933 | 0,02442 | 0,09093 | -0,37162 | 0,00000 | -0,13612 | 0,04837 | -0,23550 | 0,00088 | 0,00000 | 0,00001 | -0,16008 | 0,04963 |
| ***GALNT12*** | -0,77994 | 0,00293 | -0,83087 | 0,00027 | 0,05093 | 0,83974 | 0,00046 | 0,00684 | -1,48325 | 0,00000 | -1,12516 | 0,00001 | -0,35810 | 0,15061 | 0,00000 | 0,00000 | -0,88263 | 0,00838 |
| *GALNT13* | | | | | | | | | | | | | | | | | | |
| ***GALNT14*** | 0,41980 | 0,40693 | 0,49295 | 0,25480 | -0,07315 | 0,88475 | 0,48424 | 0,62544 | 0,92435 | 0,00016 | 1,00424 | 0,00005 | -0,07989 | 0,74322 | 0,00003 | 0,00013 | 2,02681 | 0,00000 |
| *GALNT15* | | | | | | | | | | | | | | | | | | |
| *GALNT16* | | | | | | | | | | | | | | | | | | |

Table S1

| | | | | | | | | | | | | | | | | | | |
|---|---|---|---|---|---|---|---|---|---|---|---|---|---|---|---|---|---|---|
| *GALNT17* | | | | | | | | | | | | | | | | | | |
| *GALNT18* | | | | | | | | | | | | | | | | | | |
| ***GALNT2*** | 0,22685 | 0,00768 | 0,17629 | 0,01460 | 0,05057 | 0,53999 | 0,01072 | 0,05326 | -0,07526 | 0,37841 | 0,05305 | 0,53650 | -0,12831 | 0,14168 | 0,33424 | 0,41036 | -0,16010 | 0,23758 |
| *GALNT20* | | | | | | | | | | | | | | | | | | |
| ***GALNT3*** | 0,72556 | 0,00002 | 0,55003 | 0,00014 | 0,17553 | 0,27066 | 0,00002 | 0,00091 | 0,10131 | 0,59030 | 0,29444 | 0,12084 | -0,19313 | 0,31469 | 0,29042 | 0,36578 | 1,10163 | 0,00000 |
| ***GALNT4*** | 0,18284 | 0,39647 | 0,26247 | 0,15590 | -0,07963 | 0,71117 | 0,34991 | 0,50846 | -0,13421 | 0,33983 | -0,01803 | 0,89837 | -0,11618 | 0,41751 | 0,58890 | 0,65102 | -0,03380 | 0,89125 |
| *GALNT5* | 0,88069 | 0,08725 | -0,04020 | 0,92609 | 0,92089 | 0,07410 | 0,15305 | 0,29771 | | | | | | | | | | |
| ***GALNT6*** | 0,15039 | 0,25441 | 0,02824 | 0,80079 | 0,12215 | 0,35363 | 0,50210 | 0,64000 | 0,17233 | 0,01077 | 0,08755 | 0,19397 | 0,08478 | 0,21481 | 0,03824 | 0,06547 | -0,20507 | 0,12106 |
| ***GALNT7*** | 0,03915 | 0,75967 | 0,17454 | 0,11371 | -0,13539 | 0,29236 | 0,26043 | 0,41943 | -0,28042 | 0,03362 | -0,17850 | 0,17660 | -0,10192 | 0,44594 | 0,09730 | 0,14594 | 0,33905 | 0,01773 |
| *GALNT8* | | | | | | | | | | | | | | | | | | |
| ***GALNT9*** | -0,02927 | 0,95024 | -1,10256 | 0,00761 | 1,07330 | 0,02526 | 0,01416 | 0,06382 | -0,82577 | 0,00130 | -1,09180 | 0,00003 | 0,26603 | 0,30231 | 0,00007 | 0,00024 | -0,91565 | 0,04403 |
| *GBGT1* | 0,76106 | 0,00015 | 0,29461 | 0,06964 | 0,46645 | 0,01548 | 0,00069 | 0,00900 | 0,67383 | 0,00000 | 0,56705 | 0,00000 | 0,10678 | 0,26128 | 0,00000 | 0,00000 | 0,23211 | 0,18574 |
| ***GCNT1*** | -0,05948 | 0,88286 | 0,14779 | 0,66794 | -0,20727 | 0,60803 | 0,85226 | 0,90331 | 0,33889 | 0,03198 | 0,57688 | 0,00034 | -0,23799 | 0,13809 | 0,00146 | 0,00366 | 0,36839 | 0,28709 |
| *GCNT2A* | | | | | | | | | | | | | | | | | | |
| *GCNT2B* | | | | | | | | | | | | | | | | | | |
| *GCNT2C* | | | | | | | | | | | | | | | | | | |
| *GCNT3* | | | | | | | | | | | | | | | | | | |
| *GCNT4* | -1,03547 | 0,00002 | -0,77210 | 0,00016 | -0,26337 | 0,24328 | 0,00002 | 0,00091 | -1,61738 | 0,00000 | -1,20842 | 0,00000 | -0,40896 | 0,05564 | 0,00000 | 0,00000 | -0,16899 | 0,39970 |
| *GCNT7* | 0,86943 | 0,02215 | 0,00105 | 0,99736 | 0,86838 | 0,02230 | 0,04113 | 0,12744 | 0,26825 | 0,26205 | 0,62191 | 0,01034 | -0,35366 | 0,14756 | 0,03657 | 0,06300 | 0,50137 | 0,31680 |
| *GLT1D1* | 0,45121 | 0,00897 | 0,42749 | 0,00394 | 0,02372 | 0,88731 | 0,00529 | 0,03366 | 0,40241 | 0,00003 | 0,54612 | 0,00000 | -0,14370 | 0,13145 | 0,00000 | 0,00000 | 0,77709 | 0,00004 |
| *GLT6D1* | | | | | | | | | | | | | | | | | | |
| *GLT8D1* | -0,19008 | 0,04285 | -0,09709 | 0,21968 | -0,09299 | 0,31500 | 0,11749 | 0,25065 | -0,10275 | 0,08856 | -0,04855 | 0,42172 | -0,05420 | 0,37661 | 0,23334 | 0,30490 | -0,08250 | 0,34526 |
| *GLT8D2* | | | | | | | | | | | | | | | | | | |
| *GTDC1* | 0,08022 | 0,56028 | 0,15894 | 0,17890 | -0,07872 | 0,56762 | 0,40192 | 0,55583 | -0,23738 | 0,06753 | -0,00584 | 0,96414 | -0,23154 | 0,08018 | 0,11888 | 0,17297 | 0,24625 | 0,17414 |
| *GXYLT1* | 0,16370 | 0,15606 | 0,11572 | 0,23836 | 0,04798 | 0,67505 | 0,29769 | 0,45775 | -0,10013 | 0,44345 | 0,02888 | 0,82583 | -0,12901 | 0,33313 | 0,59361 | 0,65575 | 0,38285 | 0,00714 |
| *GXYLT2* | | | | | | | | | | | | | | | | | | |
| *GYG1* | 0,82890 | 0,00001 | 0,79415 | 0,00000 | 0,03474 | 0,83818 | 0,00000 | 0,00010 | 1,04425 | 0,00000 | 1,07054 | 0,00000 | -0,02628 | 0,86439 | 0,00000 | 0,00000 | 1,38226 | 0,00000 |
| *GYG2* | | | | | | | | | | | | | | | | | | |
| *GYS1* | 0,11629 | 0,30064 | -0,13489 | 0,16090 | 0,25118 | 0,02798 | 0,07778 | 0,19159 | 0,01299 | 0,87268 | 0,10654 | 0,19222 | -0,09355 | 0,25862 | 0,36916 | 0,44606 | -0,65074 | 0,00013 |
| *GYS2* | | | | | | | | | | | | | | | | | | |
| *HAS1* | 0,89736 | 0,12454 | 0,94852 | 0,05834 | -0,05116 | 0,92948 | 0,12051 | 0,25534 | 0,20446 | 0,48313 | 0,07302 | 0,80312 | 0,13144 | 0,65822 | 0,77730 | 0,81775 | 0,88161 | 0,11600 |
| *HAS2* | | | | | | | | | | | | | | | | | | |

| | | | | | | | | | | | | | | | | | | |
|---|---|---|---|---|---|---|---|---|---|---|---|---|---|---|---|---|---|---|
| *HAS3* | -0,52656 | 0,03345 | -0,32912 | 0,11565 | -0,19744 | 0,41703 | 0,07950 | 0,19417 | -0,44920 | 0,01264 | -0,17919 | 0,31823 | -0,27001 | 0,13891 | 0,04289 | 0,07228 | -0,40689 | 0,18072 |
| *KDELC1* | 0,69494 | 0,12715 | -0,50383 | 0,19340 | 1,19876 | 0,00989 | 0,03424 | 0,11344 | | | | | | | | | 0,52565 | 0,37298 |
| *KDELC2* | -0,15081 | 0,43738 | -0,28813 | 0,08520 | 0,13731 | 0,47931 | 0,22417 | 0,37943 | -1,02176 | 0,00001 | -0,64918 | 0,00375 | -0,37258 | 0,09789 | 0,00003 | 0,00011 | -0,67470 | 0,00662 |
| *LARGE1* | -0,72544 | 0,02141 | -1,14312 | 0,00005 | 0,41768 | 0,17834 | 0,00024 | 0,00443 | -1,07267 | 0,00000 | -1,11124 | 0,00000 | 0,03857 | 0,83453 | 0,00000 | 0,00000 | -1,65573 | 0,00001 |
| *LARGE2* | -0,86330 | 0,00189 | -1,03920 | 0,00002 | 0,17589 | 0,50891 | 0,00006 | 0,00184 | -1,06741 | 0,00000 | -0,93003 | 0,00000 | -0,13738 | 0,35432 | 0,00000 | 0,00000 | -1,91056 | 0,00000 |
| *LFNG* | -0,17197 | 0,15284 | -0,35108 | 0,00101 | 0,17911 | 0,13679 | 0,00429 | 0,02942 | -0,26296 | 0,00006 | -0,34405 | 0,00000 | 0,08109 | 0,21423 | 0,00000 | 0,00000 | -0,45281 | 0,00187 |
| *MFNG* | -0,39584 | 0,00563 | -0,17469 | 0,14173 | -0,22115 | 0,11322 | 0,02017 | 0,08009 | -0,18479 | 0,02191 | -0,21722 | 0,00754 | 0,03243 | 0,69076 | 0,01483 | 0,02873 | -0,49109 | 0,00675 |
| *MGAT1* | 0,49301 | 0,00020 | 0,26349 | 0,01554 | 0,22951 | 0,06899 | 0,00069 | 0,00897 | 0,41103 | 0,00000 | 0,38495 | 0,00000 | 0,02609 | 0,73837 | 0,00000 | 0,00000 | 0,05620 | 0,64662 |
| *MGAT2* | 0,02521 | 0,81134 | 0,02630 | 0,77035 | -0,00109 | 0,99180 | 0,95025 | 0,96758 | -0,23080 | 0,01265 | -0,21026 | 0,02358 | -0,02054 | 0,82595 | 0,02171 | 0,03997 | 0,05728 | 0,51380 |
| *MGAT3* | 0,06170 | 0,83397 | -0,20067 | 0,42531 | 0,26237 | 0,37437 | 0,60361 | 0,72165 | -0,46334 | 0,00191 | -0,44138 | 0,00322 | -0,02195 | 0,88351 | 0,00215 | 0,00520 | -0,41974 | 0,06022 |
| *MGAT4A* | -0,18677 | 0,46419 | -0,44581 | 0,04378 | 0,25905 | 0,31106 | 0,12718 | 0,26369 | -0,61144 | 0,00000 | -0,60202 | 0,00001 | -0,00941 | 0,94304 | 0,00000 | 0,00001 | -0,16008 | 0,39529 |
| *MGAT4B* | 0,09660 | 0,41367 | 0,11481 | 0,25594 | -0,01821 | 0,87717 | 0,48788 | 0,62853 | 0,35982 | 0,00000 | 0,28059 | 0,00016 | 0,07923 | 0,28329 | 0,00000 | 0,00001 | 0,08204 | 0,47752 |
| *MGAT4C* | | | | | | | | | | | | | | | | | | |
| *MGAT4D* | | | | | | | | | | | | | | | | | | |
| *MGAT5* | 0,16110 | 0,67370 | -0,29350 | 0,36971 | 0,45460 | 0,23733 | 0,45055 | 0,59742 | -0,13334 | 0,42342 | -0,19589 | 0,24261 | 0,06255 | 0,71245 | 0,48629 | 0,55727 | -0,35913 | 0,26810 |
| *MGAT5B* | | | | | | | | | | | | | | | | | | |
| *OGT* | -0,12994 | 0,21563 | -0,11411 | 0,20249 | -0,01583 | 0,87925 | 0,32929 | 0,48892 | -0,46150 | 0,00001 | -0,28649 | 0,00534 | -0,17501 | 0,09075 | 0,00004 | 0,00016 | -0,02626 | 0,83836 |
| *PIGA* | 0,46333 | 0,00288 | -0,04771 | 0,70805 | 0,51104 | 0,00111 | 0,00260 | 0,02120 | -0,36101 | 0,01544 | -0,34913 | 0,01972 | -0,01188 | 0,93711 | 0,02234 | 0,04097 | -0,55898 | 0,07700 |
| *PIGB* | -0,05444 | 0,70939 | -0,01714 | 0,89048 | -0,03730 | 0,79842 | 0,93207 | 0,95621 | -0,06030 | 0,58448 | 0,10109 | 0,36216 | -0,16139 | 0,15231 | 0,35037 | 0,42692 | 0,22396 | 0,09210 |
| *PIGM* | 0,11014 | 0,37324 | -0,04467 | 0,67111 | 0,15480 | 0,21232 | 0,45290 | 0,59955 | 0,14484 | 0,05833 | -0,09478 | 0,21626 | 0,23962 | 0,00234 | 0,00890 | 0,01832 | -0,00415 | 0,97468 |
| *PIGV* | 0,01527 | 0,92773 | -0,07298 | 0,61171 | 0,08825 | 0,60064 | 0,82865 | 0,88772 | 0,07974 | 0,34680 | 0,13640 | 0,11033 | -0,05667 | 0,51167 | 0,27335 | 0,34780 | -0,06639 | 0,69731 |
| *PIGZ* | -0,23703 | 0,33751 | -0,22565 | 0,28480 | -0,01137 | 0,96314 | 0,48164 | 0,62350 | -0,18112 | 0,25522 | -0,23346 | 0,14513 | 0,05234 | 0,74662 | 0,30534 | 0,38109 | -0,84574 | 0,01852 |
| *POFUT1* | -0,01037 | 0,90727 | -0,07932 | 0,29868 | 0,06895 | 0,44007 | 0,54324 | 0,67419 | -0,22736 | 0,00033 | -0,17672 | 0,00516 | -0,05065 | 0,42395 | 0,00080 | 0,00215 | -0,17432 | 0,08510 |
| *POFUT2* | 0,02676 | 0,76533 | -0,19787 | 0,01188 | 0,22462 | 0,01470 | 0,01421 | 0,06394 | -0,28936 | 0,00018 | -0,12275 | 0,10715 | -0,16661 | 0,03164 | 0,00085 | 0,00227 | -0,13277 | 0,17111 |
| *POGLUT1* | -0,27441 | 0,01086 | -0,13205 | 0,14262 | -0,14236 | 0,17703 | 0,03528 | 0,11588 | -0,51648 | 0,00069 | -0,65007 | 0,00003 | 0,13359 | 0,38079 | 0,00005 | 0,00017 | -0,12567 | 0,46143 |
| *POMGNT1* | -0,20963 | 0,08982 | -0,30442 | 0,00477 | 0,09479 | 0,43831 | 0,01600 | 0,06910 | -0,39758 | 0,00000 | -0,37189 | 0,00001 | -0,02569 | 0,75384 | 0,00000 | 0,00000 | -0,54443 | 0,00002 |
| *POMGNT2* | -0,22279 | 0,21421 | -0,36316 | 0,01969 | 0,14037 | 0,43196 | 0,06242 | 0,16676 | -0,04101 | 0,68244 | 0,04062 | 0,68686 | -0,08162 | 0,42482 | 0,72649 | 0,77451 | -0,57633 | 0,13197 |
| *POMT1* | -0,27069 | 0,02768 | -0,42129 | 0,00012 | 0,15060 | 0,21368 | 0,00053 | 0,00749 | -0,52465 | 0,00000 | -0,51485 | 0,00000 | -0,00980 | 0,89922 | 0,00000 | 0,00000 | -0,61611 | 0,00002 |
| *POMT2* | -0,08401 | 0,52620 | -0,36856 | 0,00179 | 0,28454 | 0,03507 | 0,00543 | 0,03420 | -0,15223 | 0,00885 | -0,11309 | 0,05185 | -0,03915 | 0,50462 | 0,02412 | 0,04383 | -0,53322 | 0,00991 |
| *PYGB* | -0,08119 | 0,43026 | -0,10610 | 0,22851 | 0,02492 | 0,80825 | 0,45994 | 0,60514 | 0,05745 | 0,32100 | 0,00020 | 0,99727 | 0,05725 | 0,33204 | 0,52649 | 0,59449 | -0,32691 | 0,00360 |
| *PYGL* | 0,52776 | 0,00597 | 0,73334 | 0,00002 | -0,20558 | 0,27027 | 0,00008 | 0,00213 | 0,53541 | 0,00002 | 0,66653 | 0,00000 | -0,13113 | 0,28777 | 0,00000 | 0,00000 | 1,16892 | 0,00000 |
| *PYGM* | 0,38946 | 0,00633 | 0,28357 | 0,01867 | 0,10588 | 0,44367 | 0,01072 | 0,05326 | 0,37727 | 0,00023 | 0,46045 | 0,00001 | -0,08318 | 0,41550 | 0,00001 | 0,00005 | 0,46410 | 0,00262 |

Table S1

| | | | | | | | | | | | | | | | | | | |
|---|---|---|---|---|---|---|---|---|---|---|---|---|---|---|---|---|---|---|
| *RFNG* | 0,20747 | 0,09767 | 0,09837 | 0,35299 | 0,10909 | 0,37955 | 0,24376 | 0,40181 | 0,43644 | 0,00000 | 0,30521 | 0,00071 | 0,13123 | 0,14520 | 0,00001 | 0,00003 | -0,08208 | 0,55379 |
| *RTFDC1* | | | | | | | | | | | | | | | | | | |
| *ST3GAL1* | -0,06777 | 0,31608 | -0,02528 | 0,65980 | -0,04249 | 0,52853 | 0,60222 | 0,72060 | -0,09160 | 0,04013 | -0,09345 | 0,03729 | 0,00185 | 0,96730 | 0,05622 | 0,09135 | 0,05894 | 0,49454 |
| *ST3GAL2* | 0,58492 | 0,00637 | 0,24784 | 0,16452 | 0,33708 | 0,10796 | 0,02294 | 0,08743 | 0,09204 | 0,51882 | 0,32660 | 0,02382 | -0,23456 | 0,10808 | 0,06767 | 0,10724 | 0,63540 | 0,00279 |
| ***ST3GAL3*** | 0,14351 | 0,35845 | 0,31280 | 0,02145 | -0,16928 | 0,27942 | 0,06910 | 0,17783 | -0,05078 | 0,67122 | 0,09583 | 0,42590 | -0,14661 | 0,23037 | 0,47533 | 0,54688 | -0,07067 | 0,68927 |
| ***ST3GAL4*** | 0,32349 | 0,06340 | 0,45153 | 0,00303 | -0,12804 | 0,45657 | 0,01001 | 0,05094 | 0,51883 | 0,00001 | 0,54573 | 0,00001 | -0,02690 | 0,81878 | 0,00000 | 0,00001 | 1,10232 | 0,00000 |
| ***ST3GAL5*** | 0,15003 | 0,21775 | 0,02669 | 0,79575 | 0,12334 | 0,30988 | 0,44478 | 0,59270 | 0,12288 | 0,13498 | 0,13381 | 0,10562 | -0,01093 | 0,89587 | 0,19359 | 0,26094 | -0,02730 | 0,79938 |
| ***ST3GAL6*** | 0,18082 | 0,34995 | 0,50057 | 0,00340 | -0,31975 | 0,10116 | 0,01233 | 0,05810 | 0,25793 | 0,07166 | 0,34983 | 0,01552 | -0,09191 | 0,52711 | 0,04125 | 0,06993 | 0,95714 | 0,00000 |
| *ST6GAL1* | -0,34075 | 0,00167 | -0,36054 | 0,00014 | 0,01980 | 0,84847 | 0,00021 | 0,00415 | -0,99244 | 0,00000 | -0,73436 | 0,00004 | -0,25809 | 0,14491 | 0,00000 | 0,00000 | -0,50555 | 0,00051 |
| *ST6GAL2* | | | | | | | | | | | | | | | | | | |
| *ST6GALNAC1* | 0,22158 | 0,67886 | -0,33965 | 0,45754 | 0,56124 | 0,29634 | 0,54686 | 0,67713 | -0,82976 | 0,00809 | -0,86109 | 0,00630 | 0,03133 | 0,92098 | 0,00775 | 0,01624 | -0,96151 | 0,13216 |
| *ST6GALNAC2* | 0,23139 | 0,19682 | 0,27870 | 0,07035 | -0,04732 | 0,79036 | 0,16413 | 0,31047 | 0,39462 | 0,00066 | 0,53903 | 0,00001 | -0,14441 | 0,21414 | 0,00001 | 0,00006 | 0,42983 | 0,03822 |
| *ST6GALNAC3* | 0,64410 | 0,06872 | 0,24555 | 0,41022 | 0,39855 | 0,25566 | 0,18781 | 0,33836 | -0,05262 | 0,81534 | -0,02358 | 0,91707 | -0,02903 | 0,89942 | 0,97296 | 0,97863 | 0,26758 | 0,25200 |
| *ST6GALNAC4* | -0,00831 | 0,96919 | 0,28338 | 0,12627 | -0,29168 | 0,17851 | 0,23097 | 0,38719 | 0,52570 | 0,00012 | 0,12795 | 0,34190 | 0,39776 | 0,00400 | 0,00036 | 0,00107 | -0,00634 | 0,97250 |
| *ST6GALNAC5* | | | | | | | | | | | | | | | | | | |
| *ST6GALNAC6* | -0,31321 | 0,12231 | -0,28349 | 0,10148 | -0,02972 | 0,88220 | 0,16876 | 0,31612 | -0,60849 | 0,00006 | -0,61536 | 0,00005 | 0,00687 | 0,96353 | 0,00002 | 0,00008 | -0,49548 | 0,00547 |
| *ST8SIA1* | -0,67267 | 0,02469 | -0,99075 | 0,00020 | 0,31808 | 0,27966 | 0,00077 | 0,00960 | -0,83437 | 0,00019 | -1,06996 | 0,00000 | 0,23559 | 0,29085 | 0,00000 | 0,00002 | -0,98617 | 0,00089 |
| *ST8SIA2* | | | | | | | | | | | | | | | | | | |
| *ST8SIA3* | | | | | | | | | | | | | | | | | | |
| *ST8SIA4* | 0,30401 | 0,06459 | 0,30718 | 0,02952 | -0,00316 | 0,98443 | 0,05650 | 0,15609 | 0,07656 | 0,49780 | 0,10171 | 0,37050 | -0,02516 | 0,82694 | 0,64308 | 0,70081 | 0,71927 | 0,00001 |
| *ST8SIA5* | | | | | | | | | 0,24120 | 0,37981 | 0,39469 | 0,15368 | -0,15348 | 0,58334 | 0,35211 | 0,42845 | | |
| *ST8SIA6* | -0,08824 | 0,86553 | -1,20644 | 0,00852 | 1,11820 | 0,03544 | 0,01800 | 0,07455 | -0,50171 | 0,03160 | 0,00504 | 0,98274 | -0,50674 | 0,03321 | 0,04795 | 0,07964 | -0,88245 | 0,06967 |
| *STT3A* | -0,06812 | 0,34986 | 0,02136 | 0,73021 | -0,08948 | 0,22078 | 0,46026 | 0,60550 | -0,19648 | 0,00111 | -0,21878 | 0,00032 | 0,02230 | 0,71187 | 0,00037 | 0,00107 | -0,06501 | 0,35342 |
| *STT3B* | -0,07754 | 0,59506 | 0,00910 | 0,94165 | -0,08664 | 0,55273 | 0,82059 | 0,88177 | -0,39803 | 0,01376 | -0,18772 | 0,24410 | -0,21031 | 0,19848 | 0,04759 | 0,07916 | 0,06433 | 0,54655 |
| *UGCG* | 0,12598 | 0,29502 | 0,29860 | 0,00480 | -0,17262 | 0,15308 | 0,01772 | 0,07382 | 0,14835 | 0,29525 | 0,20510 | 0,15066 | -0,05674 | 0,69413 | 0,32754 | 0,40365 | 0,77459 | 0,00000 |
| *UGGT1* | 0,27907 | 0,05555 | 0,09864 | 0,42110 | 0,18043 | 0,21140 | 0,15732 | 0,30259 | -0,04850 | 0,52375 | -0,01248 | 0,87027 | -0,03602 | 0,64226 | 0,80446 | 0,84147 | 0,17330 | 0,07440 |
| *UGGT2* | 0,36025 | 0,14493 | 0,24613 | 0,24128 | 0,11412 | 0,64137 | 0,28598 | 0,44578 | -0,05676 | 0,74862 | 0,26095 | 0,14406 | -0,31771 | 0,07998 | 0,17381 | 0,23822 | 0,79774 | 0,00211 |
| *UGT1A1* | | | | | | | | | | | | | | | | | | |
| *UGT1A10* | | | | | | | | | | | | | | | | | | |
| *UGT1A3* | | | | | | | | | | | | | | | | | | |
| *UGT1A4* | | | | | | | | | | | | | | | | | | |
| *UGT1A5* | | | | | | | | | | | | | | | | | | |

Table S1

| | | | | | | | | | | | | | | | | | | |
|---|---|---|---|---|---|---|---|---|---|---|---|---|---|---|---|---|---|---|
| *UGT1A6* | | | | | | | | | | | | | | | | | | |
| *UGT1A7* | | | | | | | | | | | | | | | | | | |
| *UGT1A8* | | | | | | | | | | | | | | | | | | |
| *UGT1A9* | | | | | | | | | | | | | | | | | | |
| *UGT2A1* | | | | | | | | | | | | | | | | | | |
| *UGT2A3* | | | | | | | | | | | | | | | | | | |
| *UGT2B10* | | | | | | | | | | | | | | | | | | |
| *UGT2B11* | -0,27421 | 0,49969 | -0,86615 | 0,01476 | 0,59194 | 0,14808 | 0,04578 | 0,13632 | | | | | | | | | -0,01751 | 0,97413 |
| *UGT2B15* | | | | | | | | | | | | | | | | | | |
| *UGT2B17* | | | | | | | | | | | | | | | | | | |
| *UGT2B28* | 0,20101 | 0,61911 | -1,09919 | 0,00223 | 1,30020 | 0,00206 | 0,00157 | 0,01507 | | | | | | | | | | |
| *UGT2B4* | | | | | | | | | | | | | | | | | | |
| *UGT2B7* | | | | | | | | | | | | | | | | | | |
| *UGT3A1* | | | | | | | | | | | | | | | | | | |
| *UGT3A2* | | | | | | | | | | | | | | | | | | |
| *UGT8* | -1,17815 | 0,00178 | -0,83664 | 0,00840 | -0,34151 | 0,34558 | 0,00297 | 0,02309 | -0,47266 | 0,01870 | -0,05550 | 0,78167 | -0,41716 | 0,04136 | 0,03885 | 0,06639 | -2,21540 | 0,00002 |
| *WBSCR17* | | | | | | | | | | | | | | | | | | |
| *XXYLT1* | 0,00377 | 0,97845 | 0,02204 | 0,85291 | -0,01828 | 0,89569 | 0,98117 | 0,98668 | -0,03448 | 0,65909 | -0,01425 | 0,85606 | -0,02023 | 0,79954 | 0,90639 | 0,92529 | -0,70713 | 0,00396 |
| *XYLT1* | -0,22064 | 0,03769 | -0,26315 | 0,00430 | 0,04251 | 0,68323 | 0,01111 | 0,05442 | -0,79912 | 0,00000 | -0,60452 | 0,00000 | -0,19460 | 0,09100 | 0,00000 | 0,00000 | -0,58810 | 0,00012 |
| *XYLT2* | -0,16348 | 0,07884 | -0,27935 | 0,00070 | 0,11587 | 0,20974 | 0,00296 | 0,02306 | -0,16621 | 0,00423 | -0,13673 | 0,01872 | -0,02947 | 0,61430 | 0,00920 | 0,01888 | -0,61382 | 0,00001 |

*** Abbreviations:** FC, Fold Change**;** FDR, False Discovery Rate

** Bold indicates the genes encoding enzymes participating on the $sLe^x$ biosynthetic pathway

*** Genes with expression levels below the threshold in the datasets


[#] Singhania, A., R. Verma, C.M. Graham, J. Lee, T. Tran, M. Richardson, P. Lecine, P. Leissner, M.P.R. Berry, R.J. Wilkinson, K. Kaiser, M. Rodrigue, G. Woltmann, P. Haldar, and A. O'Garra. 2018. A modular transcriptional signature identifies phenotypic heterogeneity of human tuberculosis infection. *Nat Commun* 9:2308.

**Table S2. Oligonucleotide sequences used in this study**.

| Gene | Forward (5´-3´) | Reverse (5´-3´) |
|---|---|---|
| *Tnf* | GCCACCACGTCTTCTGTCT | TGAGGGTCTGGGCCATAGAAC |
| *Ifng* | CCTGGTGCACAACATCGCC | AATCAAACAGAGGCCGCATGC |
| *Il17* | CTCAGACTACCTCAACCGTTCCA | TTCCCTCCGCATTGACACA |
| *Il10* | ATTTGAATTCCCTGGGTGAGAAG | CACAGGGGAGAAATCGATGACA |
| *Ifnb* | GCACTGGGTGGAATGAGACT | AGTGGAGAGCAGTTGAGGACA |
| *Il1a* | GGGAAGATTCTGAAGAAGAG | GAGTAACAGGATATTTAGAGTCG |
| *Il1b* | ACCTTCCAGGATGAGGACATGA | AACGTCACACACCAGCAGGTTA |
| *Il12* | CAAATTACTCCGGACGGTTC | AGAGACGCCATTCCACATGTC |
| *Nos2* | CTGGGAGCGCTCTAGTGAAG | CGATGCACAACTGGGTGAAC |
| *Cxcl1* | AGCTTGAAGGTGTTGCCCTCAG | AGCTTCAGGGTCAAGGCAAGC |
| *Cxcl2* | CCCAGACAGAAGTCATAGCCAC | CTTCCGTTGAGGGACAGCAG |
| *Cxcl5* | GAGCTGCGTTGTGTTTGCTT | TAGCTTTCTTTTTGTCACTGCCC |
| *Elane* | ATGTCAGCAGCCCACTGTGT | ATCCGCTGCACAGAGAAGGT |
| *Ncf1* | CTATCTGGAGCCCCTTGACA | ACAGGGACATCTCGTCCTCTT |
| *Mmp8* | TGGTGATTTCTTGCTAACCCC | TACACTCCAGACGTGAAAAGC |
| *Mmp9* | GGACCCGAAGCGGACATTG | CGTCGTCGAAATGGGCATCT |
| *Mpo* | TGTCCGTGTCAAGTGGCTGT | AGTGGGGCTTCGTCTGTTGT |
| *S100a8* | TGAGTGCCTCAGTTTGT | CCAACACAAGGAACTCTTCG |
| *Ubiquitin* | TGGCTATTAATTATTCGGTCTGCAT | GCAAGTGGCTAGAGTGCAGAGTAA |

**Table S3. Antibodies used in this study.**

| Flow cytometry - Human blood | | |
|---|---|---|
| **Target** | **Clone** | **Company** |
| CD3 | UCHT1 | BD Pharmigen |
| CD14 | 61D3 | Invitrogen |
| CD15s | CSLEX1 | BD Pharmigen |
| CD19 | HIB19 | eBioscience |
| CD16 | eBioCB16 | eBioscience |
| **Flow cytometry - Mouse blood, bone marrow and lung** | | |
| **Target** | **Clone** | **Company** |
| CD3 | 145-2C11 | eBioscience |
| CD4 | RM4-5 | eBioscience |
| CD8 | 5H10-1 | Biolegend |
| CD11b | M1/70 | Biolegend |
| CD11c | N418 | Biolegend |
| CD19 | eBio1D3 | eBioscience |
| CD45 | 30-F11 | Biolegend |
| CXCR4 | L276F12 | Biolegend |
| Ly6C | AL-21 | BD Pharmingen |
| Ly6G | 1A8 | Biolegend |
| **Flow cytometry - Mouse bone marrow precursors (Lin$^+$)** | | |
| **Target** | **Clone** | **Company** |
| B220 | RA3-6B2 | Biolegend |
| CD3 | 145-2C11 | Biolegend |
| CD4 | A161A1 | Biolegend |
| CD8 | 53-6.7 | Biolegend |
| CD11b | M1/70 | Biolegend |
| CD11c | HL3 | Biolegend |
| CD19 | 6D5 | Biolegend |
| Gr1 | RB6-8C5 | Biolegend |
| NK1.1 | PK136 | Biolegend |
| Ter119 | TER119 | Biolegend |

| Flow cytometry - Mouse bone marrow progenitors (Lin$^-$) | | |
|---|---|---|
| **Target** | **Clone** | **Company** |
| CD34 | RAM34 | eBioscience |
| c-Kit | 2B8 | Biolegend |
| FcγR | 24G2 | BD Pharmigen |
| Flt3 | A2F10 | Biolegend |
| IL-7Rα | A7R34 | Biolegend |
| Sca-1 | E13-161.7 | BD Pharmigen |
| **Intravenous injection / Flow cytometry - Mouse bone marrow** | | |
| **Target** | **Clone** | **Company** |
| CD45 (PE conjugated) | 30-F11 | Biolegend |
| **Intraperitoneal injection - Neutrophils depletion** | | |
| **Target** | **Clone** | **Company** |
| Ly6G | 1A8 | BioXcell |
| **Immunofluorescence – Mouse lung** | | |
| **Target** | **Clone** | **Company** |
| MPO | AF3667 | R&D |
| Anti-goat | A-11057 | Invitrogen |
| **Immunohistochemistry – Human lung** | | |
| **Target** | **Clone** | **Company** |
| CD15s (sLe$^x$) | CSLEX1 | BD Pharmigen |
| Anti-mouse | E0354 | Dako |

Table S3
